# Deep Learning to Analyze Sliding Drops

**DOI:** 10.1021/acs.langmuir.2c02847

**Published:** 2023-01-12

**Authors:** Sajjad Shumaly, Fahimeh Darvish, Xiaomei Li, Alexander Saal, Chirag Hinduja, Werner Steffen, Oleksandra Kukharenko, Hans-Jürgen Butt, Rüdiger Berger

**Affiliations:** Max Planck Institute for Polymer Research, Ackermannweg 10, D-55128 Mainz, Germany

## Abstract

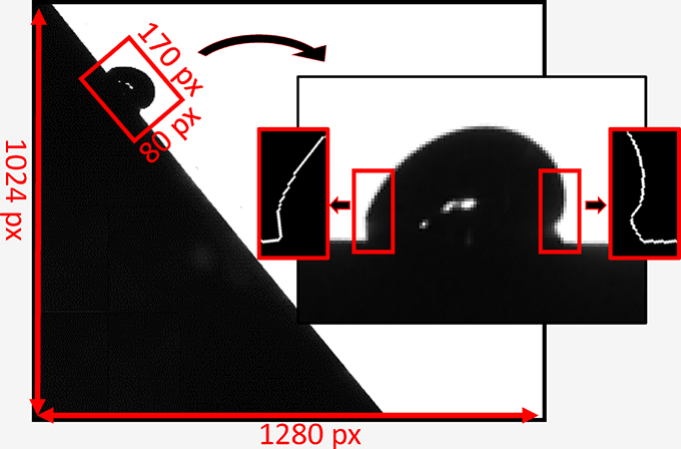

State-of-the-art contact angle measurements usually involve image analysis of sessile drops. The drops are symmetric and images can be taken at high resolution. The analysis of videos of drops sliding down a tilted plate is hampered due to the low resolution of the cutout area where the drop is visible. The challenge is to analyze all video images automatically, while the drops are not symmetric anymore and contact angles change while sliding down the tilted plate. To increase the accuracy of contact angles, we present a 4-segment super-resolution optimized-fitting (4S-SROF) method. We developed a deep learning-based super-resolution model with an upscale ratio of 3; i.e., the trained model is able to enlarge drop images 9 times accurately (PSNR = 36.39). In addition, a systematic experiment using synthetic images was conducted to determine the best parameters for polynomial fitting of contact angles. Our method improved the accuracy by 21% for contact angles lower than 90° and by 33% for contact angles higher than 90°.

## Introduction

Sessile drops on solid surfaces assume a semispherical shape to attain a state of minimal energy.^1−4^ The shape of sessile drops is axisymmetric, and it can be fitted with a solution of the Laplace equation.^5−7^ In a real wetting situation, the contact line is trapped in a metastable state. The contact angle (CA) lies in a range between the advancing CA (θ_a_) and the receding CA (θ_r_), depending on how the drop is placed on the surface.^8^ Therefore, the θ_a_ and θ_r_ are used as representative parameters that describe substrate wettability and surface tension.^9,10^ The θ_a_ represents the angle at which the liquid advances over a solid surface and the θ_r_ is the angle of a receding contact line.^11^ The CAs extracted are apparent CAs between the solid–liquid and the liquid–air interfaces at the contact line. The difference between both angles is called CA hysteresis.^12^

When drops move, for example, down a tilted plate, they are not axisymmetric anymore. They become more and more elongated depending on velocity. In addition, the CAs become dynamic CAs. For simplicity, we keep the terms advancing and receding CAs. At the front side, they assume the θ_a_, and at the rear side, the θ_r_.

**Figure 1:**
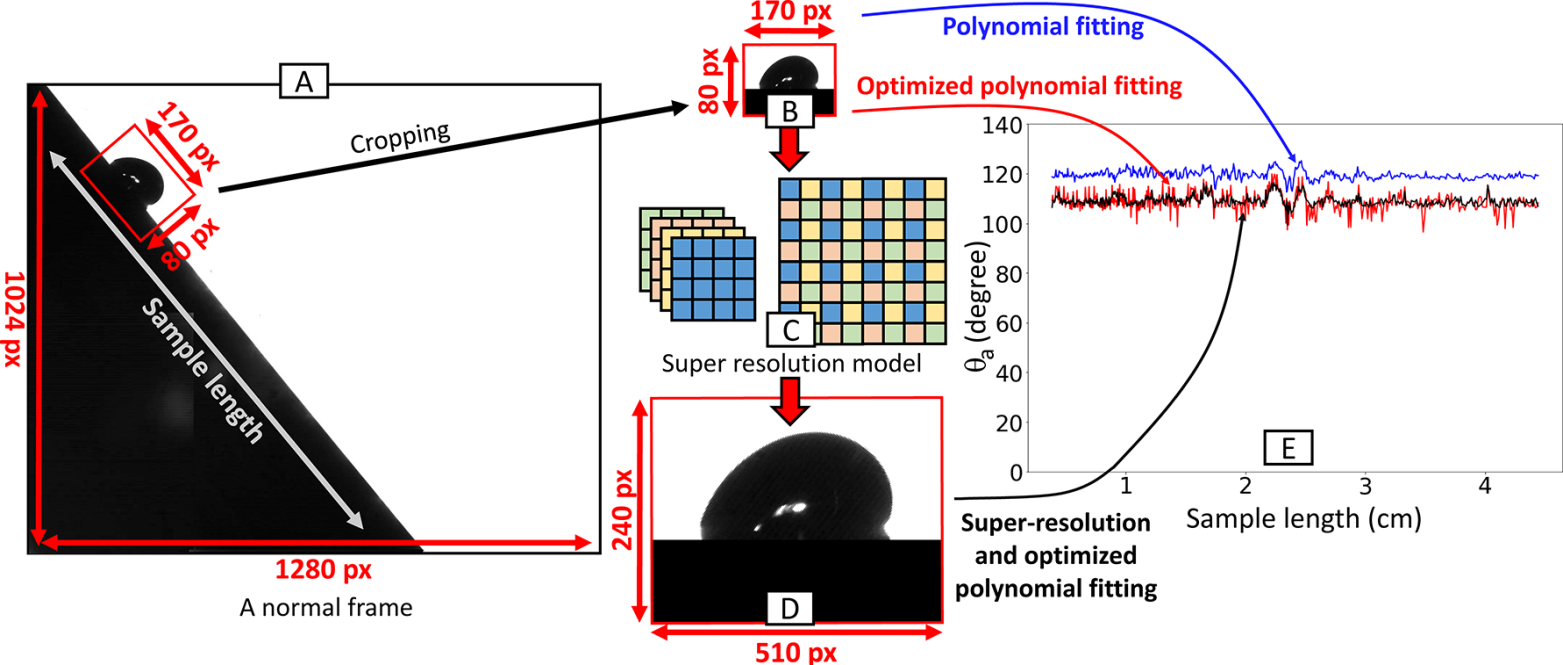
Example snapshot from a recorded video and processing routine presented in this article. (A) Full image resolution (1280 by 1024 pixels). (B) Original resolution of the extracted drop image (170 by 80 pixels). (C) Illustration of the applied super-resolution model. (D) Drop image with increased resolution (240 by 510 pixels). (E) Comparison of the calculated advancing angle on the original image with polynomial fitting (blue), with the optimized polynomial fitting presented in this paper (red), on the drop with the optimized fitting on the super-resolution image (black).

A single drop takes up just a tiny part of each video frame since the entire slide path of a drop must be recorded. Focusing with a higher magnification objective on the droplet in a specific position would result in a loss of information for the trajectory of the sliding drop. As a result, the resolution at which the drop contour and three-phase contact line can be resolved is limited by the pixel size. In a typical example, the captured image has a dimension of 1280 by 1024 pixels, while the average dimension of the drop is only 170 by 80 pixels (Figure 1A). Thus, the first and most important challenge in analyzing sliding drops on a tilted plate is to enhance resolution (Figure 1B–D). Extracting CAs can be more accurate using super-resolution images (Figure 1E).

For a sliding drop, the θ_a_ usually increases, while the θ_r_ decreases with the increase of velocity. Therefore, a method that calculates θ_a_ and θ_r_ separately is required in order to measure dynamic CAs. Based on the Weierstrass approximation theorem, polynomials can be used to approximate uniformly any continuous function of a single variable defined on a closed interval.^13^ Researchers have demonstrated that accurate CAs can be obtained for symmetric and asymmetric situations using polynomial fitting.^14,15^ However, a wide range of CAs cannot be calculated with a specific polynomial order or a predetermined number of pixels of the drop surface.^16^ It means that the order of the polynomial and the number of input pixels are two crucial parameters to obtain accurate CAs. So far, the best results have been reported for polynomial order ranging between two and four.^14,15,17^ Most reports, however, lack a proper analysis of standards or references to estimate the accuracy. Correlation coefficients and standard deviations of the results were given to evaluate the quality of fitting. Those errors based on reproducibility without existing standard reference may lead to systematic errors in measuring CAs. For asymmetric drops, the error can be up to 5° using polynomial fitting approaches^15^ and they are sensitive to drop image resolution. Methods based on polynomials can measure the CAs of both sides of the drop independently. In practice, however, the implementation is complicated because parameters need to be extracted based on the problem conditions. Here, we suggest a method for quantitative estimation of fitting errors based on the analytical expressions of artificially generated reference drops. This approach will be used to extract polynomial parameters based on the optimization of the fitting errors. The accuracy of fitting methods is highly dependent on the image resolution.

Recovering a high-resolution image or video from its low-resolution counterpart is an active area in digital image processing.^18^ It is referred to as super-resolution. Super-resolution can be divided into single-image super-resolution (SISR) or multi-image super-resolution (MISR). Increasing sliding drop resolution is a SISR problem. The SISR problem is ill-posed because one low-resolution image may correspond to multiple high-resolution image solutions. There are three main categories of SISR algorithms: interpolation-based, reconstruction-based, and learning-based.^19^ Interpolation-based SISR methods are fast but not very accurate. Bicubic interpolation is the most popular method in this category.^20^ Reconstruction-based methods are the second category in which sophisticated prior knowledge is the basis for these methods.^21,22^ They are slow and their accuracy is very sensitive to the scale factor. Learning-based SISR methods are attracting attention due to their high speed and accuracy. These methods are based on machine learning algorithms and learn from training samples to interpret relationships between low-resolution and high-resolution images. A branch of machine learning algorithms called deep learning^23^ is able to learn informative hierarchical representations automatically. Deep learning algorithms perform better than traditional machine learning algorithms across numerous fields: deep learning algorithms in the SISR are applied, for example, in medical imaging,^24^ fluorescence microscopy in biology,^25,26^ atomic force microscopy,^27^ FIB-SEM^28^ in materials science, and reconstruction of turbulent flows in physics.^29,30^

Convolutional neural network (CNN) is one of the most successful subsets of deep learning. It is primarily used to process images.^23^ Based on CNN, many super-resolution models have been proposed.^31−34^ In all mentioned models, before reconstruction, the input image was upscaled to a high-resolution space by using a single filter, typically bicubic interpolation. In other words, the super-resolution operation takes place in the high-resolution space, which increases computational complexity substantially. To solve this problem, Shi et al.^35^ introduced the Efficient Sub-Pixel Convolutional Neural Network (ESPCN). In ESPCN, there is an efficient sub-pixel convolution layer that learns an array of upscaling filters to upscale the final low-resolution feature maps and turn them into the high-resolution output. By replacing the handcrafted bicubic filter with a trainable layer and moving this layer to the very end of the network, they succeeded in increasing accuracy and decreasing computational complexity. Due to its efficiency and speed, ESPCN is well suited for real-time image processing and especially video analysis.

Here, we present a method to enhance the accuracy in CA determination by enhancing the resolution of video frames and by optimizing a polynomial fitting. After recording drops moving by a high-speed camera, we processed each frame of the video to extract the drop profile. Here, we faced a number of challenges: How can we increase the accuracy of CA measurements from low-resolution videos? How can we measure the CA for asymmetric and deformed drops as accurately as possible? How can we determine the improvement in the accuracy of the measured CAs (since there is no reference method for drops on a tilted plate)?

We trained an ESPCN super-resolution model with an upscale ratio of 3; i.e., the trained model was able to enlarge drop images 9 times with high accuracy. Then, we optimized a flexible polynomial fitting to measure dynamic advancing and receding CAs separately. To examine the accuracy, we conducted a systematic experiment using synthetic images.

We propose a toolkit to extract drop profiles from a high-speed camera based on a modified ESPCN super-resolution model and optimized polynomial fitting. This toolkit gains all relevant parameters such as advancing CA, receding CA, drop length, median line angle, and velocity from the videos.

## Materials and Methods

### Tilted Plate Experiments

Deionized water drops were placed on top of a tilted plate using a peristaltic pump connected to a grounded syringe needle. A high-speed camera (FASTCAM Mini UX100 (Photron) with a TitanTL telecentric lens, ×0.268, one inch, C-mount (Edmund Optics)) captured videos of sliding drops from the side view. The illumination conditions were controlled by a telecentric backlight illuminator (138 mm, Edmund Optics). Typically, the imaged slide length corresponds to 4.5 cm in all measurements. The experimental temperatures were 20 ± 1 °C and humidity levels were 15–30%.

### Sample Preparation

In this study, we performed some sliding drop experiments on hydrophobic samples with a point defect and samples with a chemical heterogeneity (a strip perpendicular to the sliding direction). For a better understanding, the schematics of both samples are represented in Figure S1a.

#### Samples with a Topographic Defect

As substrates, 170 μm thick precision glass coverslips were used (Carl Roth no. 1.5H).^36^ Water, ethanol, and acetone were used to clean the coverslips. To prepare topographic defects, we used photolithography (masks provided by DeltaMask). The defect was a SU8 cylindrical pillar with a diameter of 1100 μm and a height of 10 μm. After cleaning the substrates in isopropanol, O_2_-plasma (Diener Electronic, Femto BLS) was used to activate the substrates for 1 min at 30 W, a flow rate of 0.3 cm^3^/s, and a pressure of 0.3 mbar. Next, the surface was exposed to the vapor of trichloro(1*H*,1*H*,2*H*,2*H*-perfluorooctyl)silane (PFOTS, SIGMA-ALDRICH Chemie GmbH, 97%). In detail, 100 μL of PFOTS liquid was placed in a desiccator (cylindrical volume, the diameter was 20 cm, and the depth was 15 cm) at 50 mbar with the samples. Samples were placed 5 cm above the PFOTS liquid container. Then, the container was placed on a magnetic stirring plate. At the end, the samples were removed from the container after 30 min.

#### Samples with a Chemical Heterogeneity

Chemically heterogeneous samples have been prepared by the process of double chemical vapor deposition with the use of a glass mask.^37^ Standard microscopic glass slides were cleaned with Milli-Q water, then with acetone, ethanol, and 5 min oxygen plasma treatment at 300 W and 0.3 bar (Diener Electronic Femto). Then, they were treated by chemical vapor deposition of PFOTS in a desiccator. PFOTS liquid (1 mL) was placed in the desiccator at less than 20 mbar for 10 min. The pump was switched off and the samples remained for 20 min. They were transferred to a vacuum oven tempered at 25 °C for another 10 min. A glass shadow mask was placed over them in an O_2_-plasma chamber (300 W, 0.3 bar) for 5 min. The uncovered portion of the PFOTS layer was obliterated with this exposure. Chemical vapor deposition began immediately in the desiccator containing 200 μL of octyltrichlorosilane (OTS, SIGMA-ALDRICH Chemie GmbH, 97%). At the end, the samples remained in the vacuum for 120 min at 150 mbar. Due to the complexity of the preparation process, a schematic of all steps is represented in Figure S1b.

### Super-Resolution Model

To increase the accuracy of θ_a_ and θ_r_, we increased image resolution using the ESPCN super-resolution model. Our input files were videos, which contained at least 200 frames of a drop in different positions. To study drop charges, researchers may need to analyze videos containing 100 subsequent drops.^38^ Thus, the calculation speed is a crucial factor for analysis of hundreds of videos in a row. We selected ESPCN because of its high processing speed and high accuracy.^35^ The dataset, ESPCN architecture and parameters, and training procedure are described below.

For training the super-resolution model, 1400 videos of sliding drops were gathered. Using a high-speed camera, subsequent frames in a video exhibit nearly identical drop shapes. It is however desirable to have different shapes of drops for training the super-resolution model. Thus, 10 frames were extracted from each video. The final dataset consisted of 14,000 images from sliding drops imaged under different conditions (Figure S2; the dataset is downloadable on GitHub^39^). The Keras^40^ and TensorFlow^41^ libraries were used to train the ESPCN model. We considered training, validation, and test sets as 80, 10, and 10%. Test set contains 1400 frames from different videos, completely separate from training and validation sets.

Before training, we needed to define the scale factor chosen to increase resolution. A scale factor of *x* indicates that the trained model enlarges drop images *x* times for each axis. The whole image is accordingly scaled by *x*^2^. In order to find out which scale factor is best, we trained three models by scale factors 2, 3, and 4. We used peak-signal-to-noise ratio (PSNR, units: dB) as a parameter to evaluate the quality of the reconstructed images.^33^ As the scale factor increases, the image resolution increases. However, the accuracy (PSNR) decreases (Figure S3). Therefore, we repeated all results presented in Section 3.2 for scale factors 2, 3, and 4. As a result, a scale factor of 3 is the best choice, especially for CAs higher than 90°.

The architecture of the ESPCN model has two layers with a depth of 64 and 32 nodes as hidden layers for feature mapping operations. The dimensions of the input images are not constant. We had widths ranging from 129 to 264 px and heights ranging from 49 to 102 px. There is another layer called the sub-pixel convolution layer to construct the super-resolution image as the output. The activation function for the ESPCN model was tanh. Reconstructed images are more precise when the PSNR is high. The PSNR was calculated by first calculating the one-dimensional mean squared error:
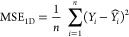
1Here, *n* is the number of data points, *Y_i_* are the observed values, and *Y_i_^* are the predicted values. To compare two matrices/images, we define the MSE_2D_ in a similar way by
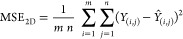
2
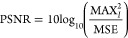
3Here, MAX_*I*_ is the maximum possible pixel value of the reference image, *Y*_(*i*, *j*)_ are the pixel values of the reference image, and *Ŷ*_(*i*, *j*)_ are the pixel values of the predicted image.

In the training process, the reference images are based on the scale factor (here is 3). Then, the super-resolution model tries to return the downsized image to the reference resolution. In this way, the model is able to compare the predicted image and the reference image and start to learn. *Y*_(*i*, *j*)_ and *Ŷ*_(*i*, *j*)_ are calculated after the training process on the test data. The model accuracy based on PSNR on 400 epochs for a scale factor of 3 was calculated at 35.46. An epoch is one cycle of training with all the training data. We have chosen 400 epochs because the training process diagram plateaued after 300 epochs (Figure S4). We modified the ESPCN architecture and added a layer with a depth of 64 nodes between the hidden layers and changed the activation function to ReLU (Figure S5). As a result of the modifications, the PSNR improved from 35.46 to 36.39 for the same scale factor and number of epochs. Since the PSNR is a logarithmic measure,^42^ differences in the order of 1 are noteworthy. In the training process of the modified ESPCN model, the training and the validation curves were compatible (Figure S4). It means that the training process has been done correctly. A common traditional model called bicubic was also used to increase the resolution and calculate related PSNR to compare it to the proposed model. The PSNR for the bicubic model was 28.90. The difference between the modified ESPCN and the bicubic model is considerable. In all cases, the modified ESPCN obtained a considerably better PSNR than bicubic (Figure S6).

**Figure 2:**
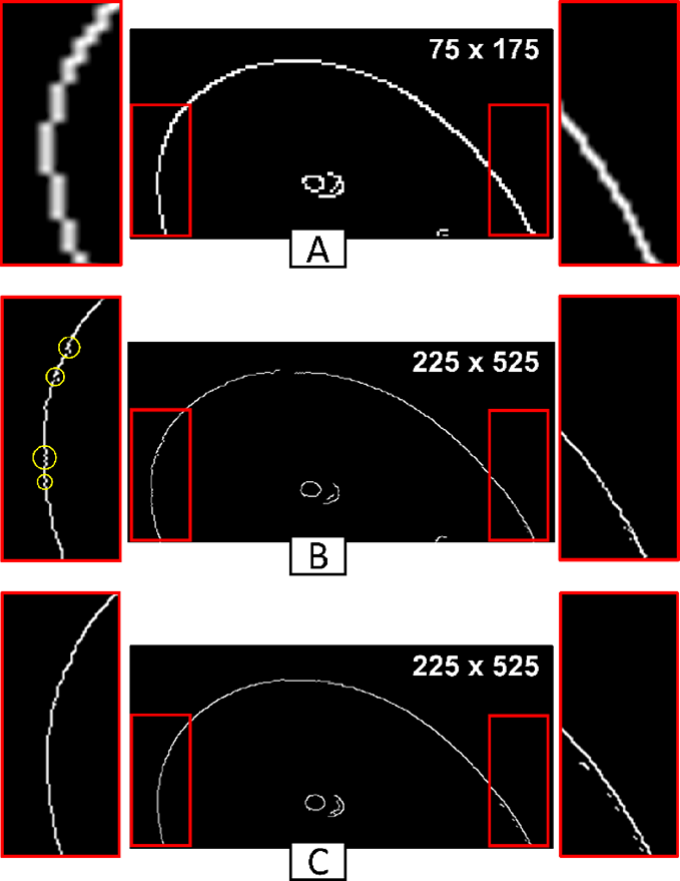
Examples of accuracies of edge detection on a drop image before using super-resolution (A), after using the bicubic method (B), and after using the super-resolution model (C). (A) The drop image in the original resolution (75 × 175 pixels). (B) The upscaled image using the bicubic method (225 × 525 pixels). The drop curve is smoother than the low-resolution image, but some edge detection displacements are visible, especially for the advancing part (yellow circles). The edge for the upper part of the drop is not detected properly, and the drop contour is not connected. (C) After applying the super-resolution model, the image has 225 × 525 pixels. Visually, the extracted edge of a super-resolution image has a very smooth curve. All mentioned drop contours were created after detecting the baseline and cropping the upside part.

We compared a representative image from a video file before and after applying the ESPCN algorithm visually and after applying the bicubic method (Figure 2). In all cases, we applied a canny edge detection algorithm^43^ implemented by OpenCV.^44^ The drop contour after application of ESPCN appears sharper with more details (Figure S7). The reason is that canny edge detection operates in a sub-pixel environment after using the super-resolution model.^35^ Increasing image details by using sub-pixel algorithms is a common approach to measuring CAs.^16,45,46^ Prior to using the super-resolution model, baseline detection is performed (details shown in the Supporting Information). Thus, the drop image was extracted without reflection before being fed into the super-resolution model.

Although the ESPCN image appears sharper, it is not clear if it will lead to a more precise extraction of the CA. To answer the question, CA improvement is discussed in the following sections.

### Polynomial Fitting

CAs can be extracted by fitting an ellipse to the drop contour when the drop is sliding smoothly.^47^ In general, the shape of a sliding drop is non-elliptic, and it is non-symmetric with respect to the front and rear. Moreover, the drop’s shape may be deformed at the advancing and receding side due to interaction with defects.^48^ Therefore, we aim to develop a universal method to calculate CAs. The polynomial fitting can be used to separately calculate θ_a_ and θ_r_ based on adjacent pixels.

**Figure 3:**
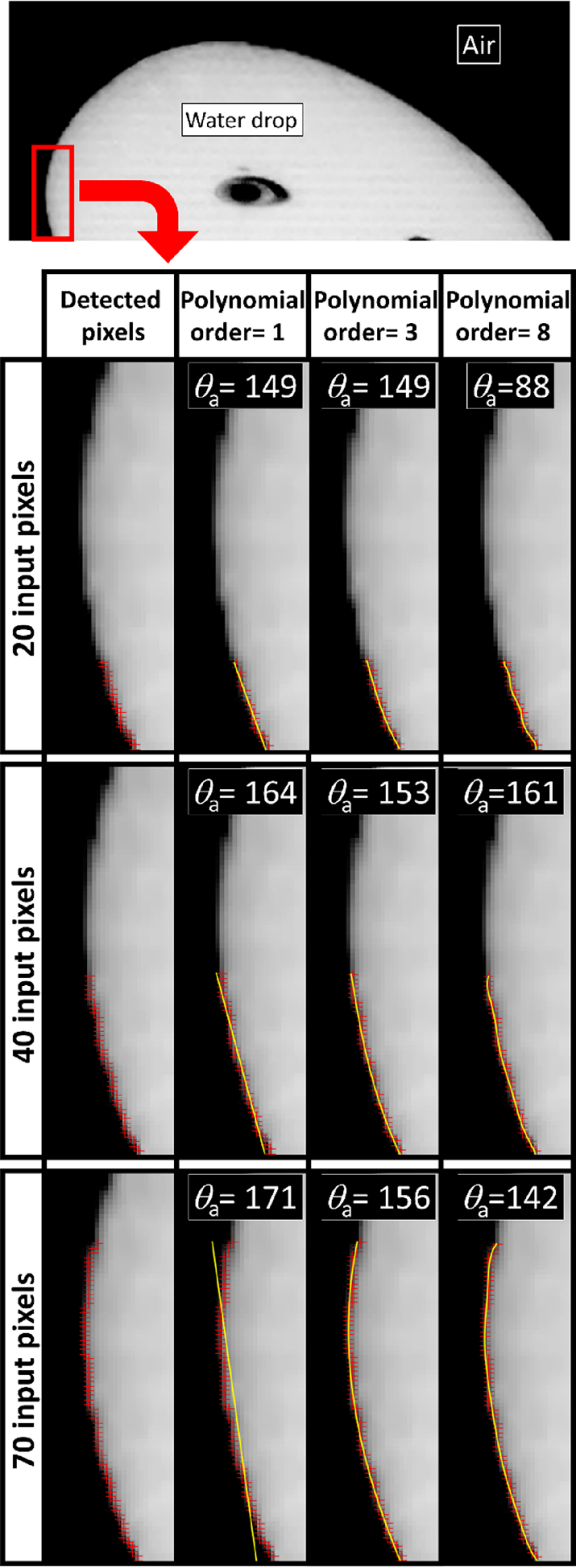
Differences in the calculated advancing angle (θ_a_) after polynomial fitting with varying parameters: numbers of pixels (20, 40, 70) and orders of the polynomial (1, 3, 8). The red plus symbols represent the selected pixels. The yellow curve represents the fitted polynomial. There is a black area in the center of the drop image that is just a reflection that appears in all recorded frames. When *p* = 1, a line is fitted to the pixels. Thus, when the number of input pixels increases, the fitting cannot follow the pixels. When *p* = 8, the polynomial follows all pixels and there is no generalization. For *p* = 3, the polynomial follows the drop shape. In this case, measurement variations for different input pixels are lower than other mentioned polynomials.

The accuracy of polynomial fitting depends on the order of the polynomial and the number of pixels taken from a contour line as input. As an example, we show how the determination of θ_a_ depends on the pixel number and the polynomial order (Figure 3). The first column shows the position of the drop contour based on the edge detection algorithm without polynomial fitting. The second column represents a fit of the drop contour with a line (polynomial order one), the third column a fit with a third-order polynomial, and the fourth column with an order of eight. For each fit, we varied the length of the contour line, i.e., 20 pixels (1st row), 40 pixels (2nd row), and 70 pixels (3rd row).

A CA with less than 20 pixels input can be approximated with a linear fit for extracting CAs, while more pixels overestimate the CA in this example. For the eighth-order polynomial, the fit follows all pixels without following a general drop contour. An appropriate polynomial order in our case can be close to the third-order polynomial, which generally follows the drop curve for all ranges of input pixels. Still, a variation in a CA of 7° results from an increase in input pixels from 20 to 70. An appropriate selection of input pixels and polynomial order can even help to reduce optical noise (Figure S8). Choosing the polynomial order that is most accurate may differ based on the circumstances of each problem. The question arises, which order of the polynomial fit function (*p*) and the number of input pixels (*n*) are required to extract the correct CA value? In addition, we expect differences for *n* and *p* for CAs <90° and CAs >90°.

## Results and Discussion

Does the ESPCN model increase the accuracy of the extracted CA? How many pixels and what polynomial order is the best for sliding drops on different surfaces? To answer these questions, a defined reference is required to calculate the accuracy of the polynomial results by changing the above variables. We use synthetic images^14^ but not containing reflections to calculate the accuracy.

### Reference Construction

**Figure 4:**
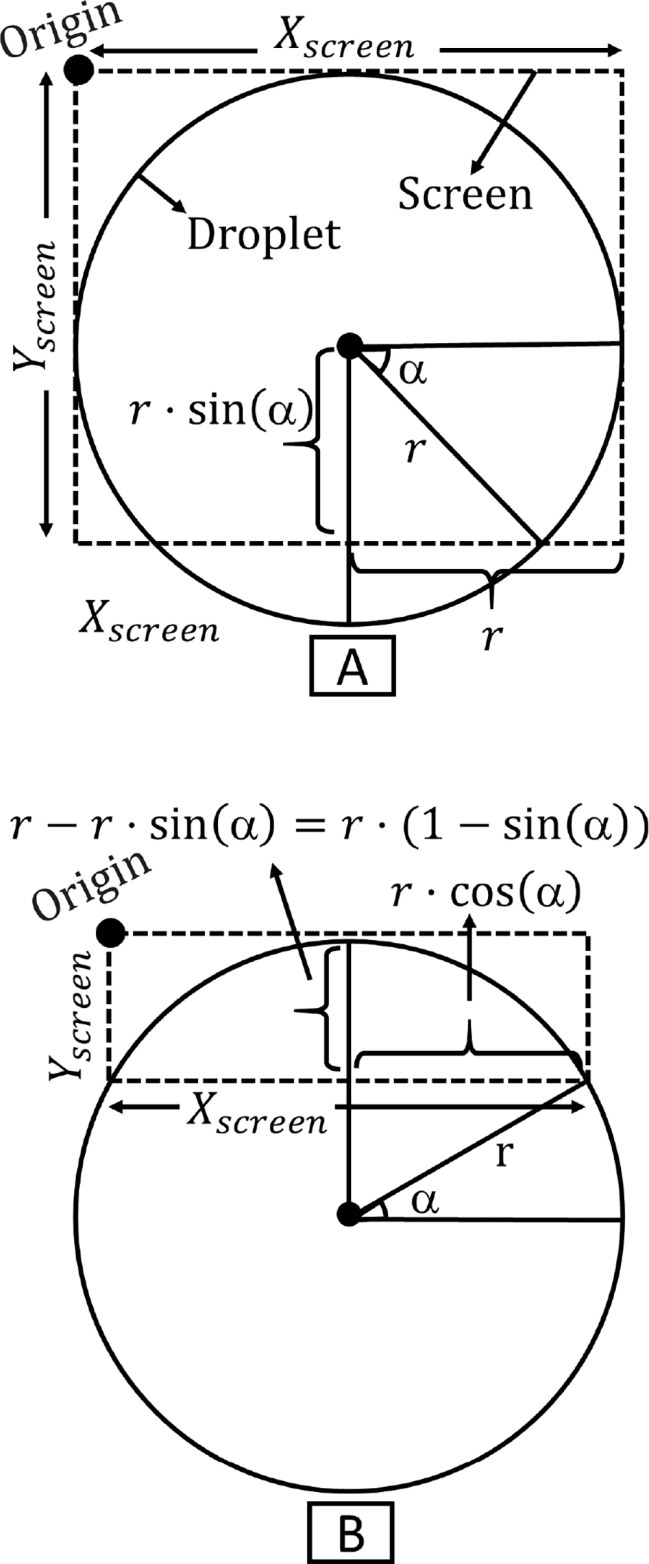
A visual representation of the components of the proposed approach for creating synthetic images. (A) To produce synthetic images for CAs >90°. (B) To produce synthetic images for CAs <90°.

A dashed box represents the screen and a circle represents a drop (Figure 4). The contour of a drop is simulated by the part of the circle that is inside the dashed box. It is possible to generate all ranges of the CAs by changing the radius of the circle and the size of the box. The images were generated in Python using the OpenCV image processing library.^44^ The *y*-axis direction in image processing is downward. Accordingly, the origin of the produced images is upside left, and the derived formulas are considered in the fourth quadrant of Cartesian coordinates.

In our case, experimental images of sliding drops extracted from videos have an average of 13,600 pixels.
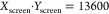
4

To match experiments, the number of pixels should be identical to the synthetic image. Therefore, eq 4 expresses the product of the vertical and horizontal number of pixels of the synthetic images.
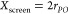
5
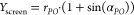
6
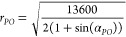
7

Equation 7 is obtained by substituting eqs 5 and 6 into eq 4. Also,  represents an equation of a circle for the hydrophobic part. Using this equation and eq 7, it is possible to generate drops with a CA higher than 90°.
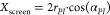
8
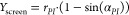
9
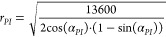
10

Equation 10 is obtained by substituting eqs 8 and 9 into eq 4. Also,  represents an equation of a circle for the hydrophilic part. Using this equation and eq 10, it is possible to generate drops with a CA lower than 90°.

PO and PI are abbreviations for hydrophobe and hydrophile. Here, *r* is the radius of the synthetic drop, and α is the angle between the horizontal line and the line that connects the center of the drop to the intersection of the circle and the box. The *Y*_screen_ is the vertical number of screen pixels, and the *X*_screen_ is the horizontal number of screen pixels. Following image generation, a Gaussian filter was applied to smooth the contour of the drop. Examples of synthetic images generated using the mentioned formulas when α was equal to 30° and 60° for CAs >90° and CAs <90° are provided in the SI (Figure S9).

We used synthetic images to simulate asymmetrical drop images by taking two halves of an image. One half represents the advancing side and the other half is the receding side of the drop. In general, θ_a_ > θ_r_ (Figure S10).

### Optimization of Polynomial Variables

MSEs were calculated for low-resolution artificial images and super-resolution images after the super-resolution model was applied. Conceptually, we distinguish hydrophilic (15–90°) and hydrophobic (90–165°) surfaces by CAs. Hereby, a calculated MSE corresponds to the related range of possible CAs for CAs <90° and CAs >90°. The MSEs (eq 1) for different polynomial orders from 1 to 10 were calculated. The number of input pixels for both low and super-resolutions starts from 5 px with a 5 px increment.

**Figure 5:**
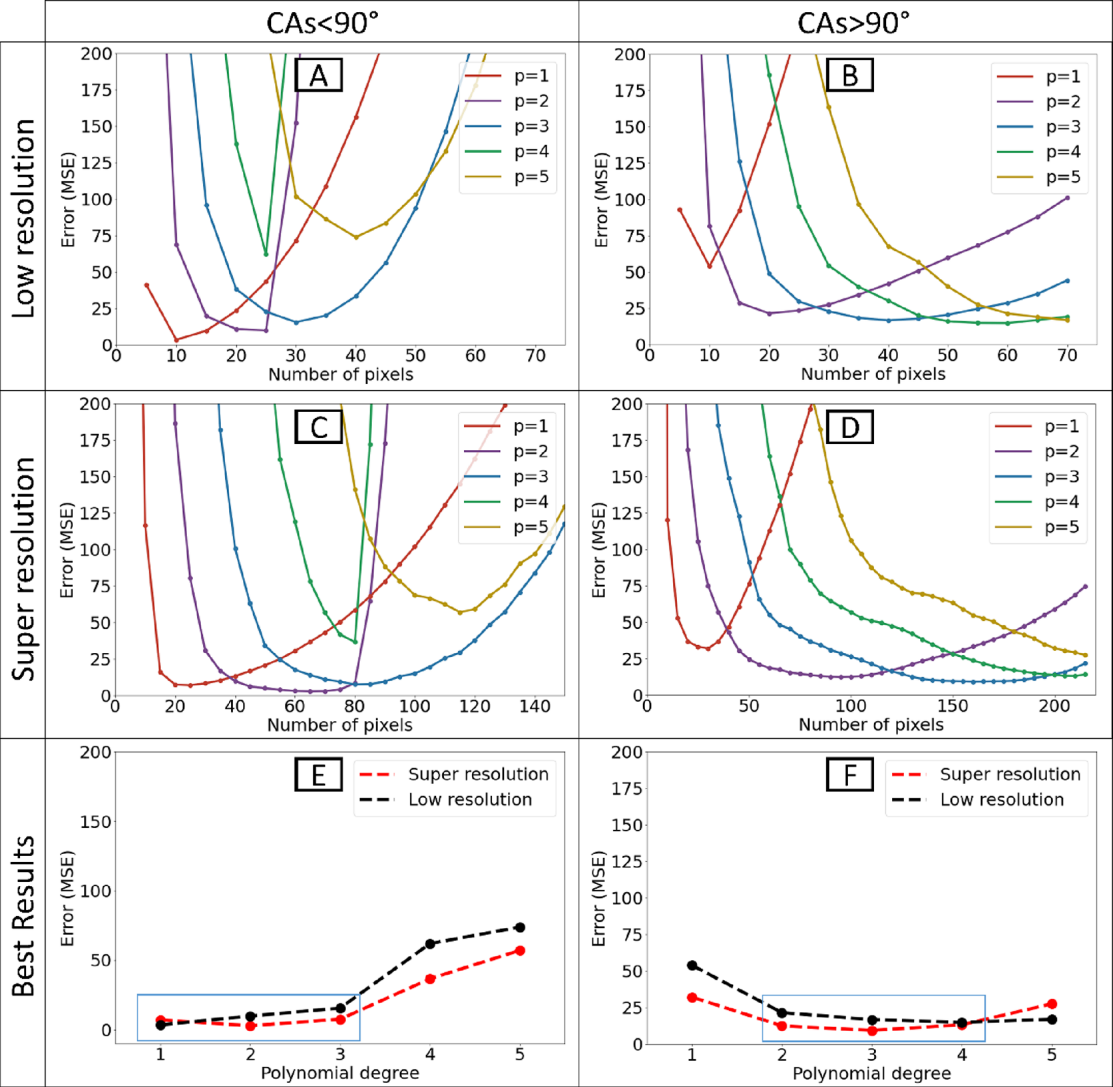
Measuring errors using low and super-resolutions and polynomial fitting with different parameters. CAs were divided into two categories: CA <90° and CA >90°. Based on eq 1, errors were calculated by comparing measured values with real values of the synthetic images. For clarity, we only plot values until the polynomial of an order 5. The error is based on the number of pixels and order of a polynomial for low-resolution images in the hydrophilic (A) and hydrophobic (B) parts and super-resolution images in the hydrophilic (C) and hydrophobic (D) parts. The best results were achieved by examining the different numbers of pixels based on each polynomial order for hydrophilic (E) and hydrophobic (F) parts.

For CAs <90° and low-resolution images, the minimum MSE is 3.41. This value corresponds to the order of a polynomial *p* = 1 and the number of input pixels *n* = 10 (Figure 5A). Thus, for hydrophilic samples, a fit of a tangent close to the three-phase contact line is the best approach. Increasing the polynomial order increases the minimal error values to 9.80, when *p* = 2, and to 15.51 for *p* = 3 (Figure 5E, black line). For CAs >90° and low-resolution images, the minimum MSE is 10.9. This error corresponds to *p* = 4 and *n* = 60 (Figure 5B). Also, *p* as 3 and 5 are good options since their MSEs are close to the minimum (Figure 5F, black line). This analysis of low-resolution images indicates that the choice of the best combination of *p* and *n* is less obvious for CAs >90°.

For CAs <90° and super-resolution images, the lowest error was 2.82, which corresponds to *p* = 2 and *n* = 65 (Figure 5C). Thus, the error of the super-resolution image is lower than the error of the tangent fitting (*p* = 1) in the low-resolution images. Taking the super-resolution images for hydrophobic samples, we obtain a minimum error of 6.28. This error value corresponds to *p* = 3 and *n* = 160 (Figure 5D). The decrease of the error from 10.9 to 6.28 indicates that the super-resolution images significantly improve the precision of the extracted CA for CAs >90°. Now, we used a binary segmentation, corresponding to CA >90° or <90°. The next step will be to investigate if a better segmentation of CAs will further decrease the error.

### CA Segmentation Optimization

**Figure 6:**
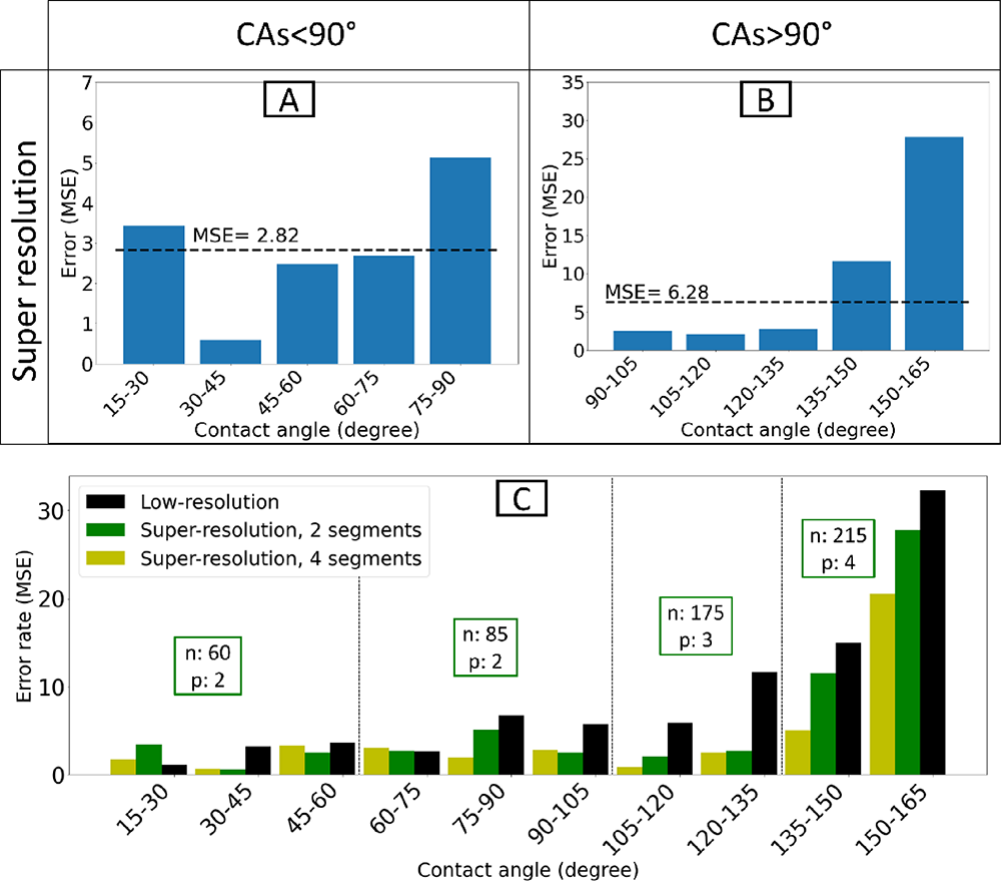
Distribution of error based on CAs. (A) The MSE for super-resolution images based on CAs from 15 to 90. The average MSE is 2.82 for *p* = 2 and *n* = 65. (B) The MSE for super-resolution images based on CAs from 90 to 165. The average MSE is 6.28 for *p* = 3 and *n* = 160. (C) New segmentation led to an improvement in MSE for both hydrophobic (4.91) and hydrophilic (2.11) parts. The segmentation and parameters in the figure are related to green bars. The yellow bars correspond to the results obtained with 2 segments and the black bars for the original low-resolution image for comparison.

Based on the prior segmentation (2 segment, from 15 to 90 and from 90 to 165), the lowest error for super-resolution images for CAs <90° was 2.82 (*p* = 2 and *n* = 65; Figure 6A) and for 6.28 (*p* = 3 and *n* = 160; Figure 6B) for CAs >90°. However, the error for 15–30, 75–90, 135–150, and 150–165 exceeds the corresponding value in the respective segments (Figure 6A,B). Hence, additionally refining these CA classes into smaller segments may further improve the error.

Using a grid search algorithm,^49^ we tested different segmentations. 4 segments improved the error (Figure 6C). CAs within 15–60° are measured when *p* = 2 and *n* = 60, 60–105° when *p* = 2 and *n* = 85, 105–135° when *p* = 3 and *n* = 175, and 135–165° when *p* = 4 and *n* = 215. To compare the accuracies, the black bars represent the results of the optimized polynomial on low-resolution images (Figure 6C). The green bars represent the 2-segment super-resolution optimized-fitting (2S-SROF) results, and the yellow bars represent the 4-segment super-resolution optimized-fitting (4S-SROF) results. Finally, the total error for >90° improved to 4.91 and for <90° improved to 2.11.

In conclusion, polynomial fitting without prior optimization can lead to a significant error. For example, if someone considers *p* = 2 and *n* = 20 (a rational choice) to determine CAs for <90° with low-resolution images, the accuracy based on MSE will be 10.76, which is more than 3 times bigger than the optimal value (3.41; *p* = 1, *n* = 10). The accuracy depends heavily on the selected *n* and *p*. The optimized polynomial fitting error before using super-resolution was 1.8° for CAs <90° and 3.3° for CAs >90°. After using the super-resolution procedure, the error decreased to 1.4° for CAs <90° and 2.2° for CAs >90° based on RMSE. We want to emphasize that this error calculation is based on our circular model and is only a subset of the shapes that a drop can take. Therefore, we prefer to report the improvements by providing percentages. The accuracy improved by 21% for CAs <90° and 33% for CAs >90° when using the 4S-SROF. We assume that the analysis of images recorded in real experiments improves by a similar percentage.

We have observed that the polynomial fitting has a shortcoming to calculate the CA for a line close to the vertical line (90°). This is because finding combinations of values for polynomial fittings that may produce a vertical line is problematic. A similar problem with using polynomials has been reported in other studies.^14^ In order to keep the CA accuracy near 90° for all calculations, we have rotated the drop boundary by 90° and calculated θ_a_ and θ_r_ while taking into account the rotation for all calculations.

## Application

### How the Toolkit Works

For our accurately known drop contours, we developed an open-source toolkit.^39^ We will briefly explain how the toolkit extracts different criteria below.

#### Right and Left Halves’ Coordinates

Consider the drop contour as two separate halves: right and left. *X*_r_ = {*x*_r1_, ..., *x*_rn_} and *Y*_r_ = {*y*_r1_, ..., *y*_rn_} are the coordinates of the right half of the drop. *X*_l_ = {*x*_l1_, ..., *x*_ln_} and *Y*_l_ = {*y*_l1_, ..., *y*_ln_} are the coordinates of the left half of the drop, and *n* is the number of drop contour pixels.

#### CAs

The first step in measuring CAs is to determine how many pixels should be selected at the front and the rear. Consider that *m* pixels from the right half and *k* pixels from the left half are needed. In this case, we will select {*x*_r1_, ..., *x*_rm_} and {*y*_r1_, ..., *y*_rm_} pixels from the right half and {*x*_l1_, ..., *x*_lk_} and {*y*_l1_, ..., *y*_lk_} from the left side. Using the extracted pixels, a polynomial fitting algorithm can be used to measure CAs.

#### Drop Length

The drop length depends on the difference between the two end pixels of the drop contour. The *X* coordinates for these two pixels are *x*_l1_ and *x*_r1_. As a result, |x_r1_ – x_l1_| represents the length of the drop. With polynomial fitting, the accuracy of determining the length of the drop increases (Figure S11).

#### Median Line Angle

*M* = {, ...,} represents the average point for each row of the drop contour. These values resemble a tilted vertical line when plotted (Figure S12). We observed that drop oscillation can be perfectly represented by the angle between the line and the horizontal line. We used its angle as a criterion. The weighted average function on *M* represents a number related to the *x* coordinate of a vertical line that can be used to divide the drop volume in half horizontally. The formula will be
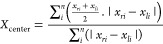
11

#### Velocity

Previously, the average velocity of the two end pixels of the drop contour was used to calculate the velocity.^50^ This method considers the middle of the drop length as the drop’s center. However, it is not representative of the velocity of other parts of the drop. Drop velocity can be measured using eq 11 since it is based on the volume distribution of the drop. Using eq 11, with respect to the camera frame rate when capturing the frames, it is possible to calculate the drop speed based on the center position of the drop in each frame.

Refer to the video in the Supporting Information for a visual representation of the extracted edge and different criteria.

**Figure 7:**
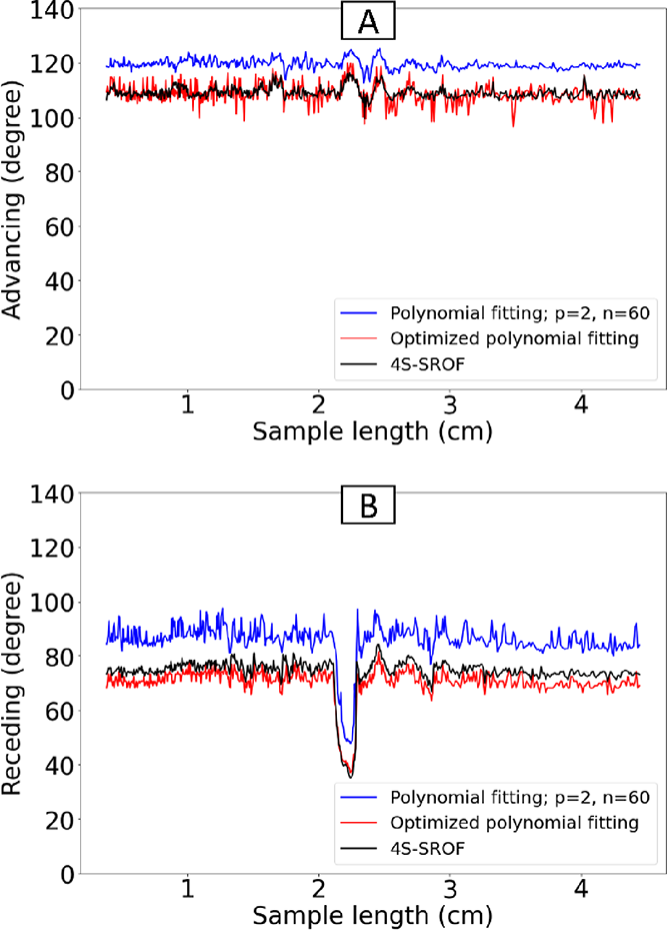
Comparison of a second-order polynomial fitting with an optimized polynomial fitting as well as a 4S-SROF for both advancing angle (A) and receding angle (B). The blue line is polynomial fitting without optimization on low-resolution images for *p* = 2 and *n* = 60. The red line is polynomial fitting after optimization on low-resolution images for *p* = 3 and *n* = 40. The black line is polynomial fitting after optimization on super-resolution images (4S-SROF).

We applied the above toolkit to investigate a sample with a defect. We recorded high-speed videos of 35 μL drops of deionized water sliding down tilted surfaces. The tilt angle was 50°. The hydrophilic round defect had a diameter of 1100 μm. We applied optimized polynomial fits on low-resolution images and the above discussed 4S-SROF and extracted θ_a_ and θ_r_ (Figure 7). In addition, we compared the CAs with the ones calculated by a second-order polynomial fit, which is frequently reported in the literature.^14,15^ Both the optimized polynomial fits and 4S-SROF lead to CAs that are 108° (Figure 7A) and 75° (Figure 7B). In addition, the 4S-SROF fits result in reduced fluctuations of the signals. The limited number of available data points (*n*) in low-resolution images causes the fluctuations. Here, super-resolution reduces fluctuations and thus increases accuracy due to more available data points. As a result, the measurement of CAs >90° is greatly improved by the super-resolution method (Figure 7A). In the 4S-SROF, the trend will be more obvious. It is due to improved accuracy when calculating super-resolution images (Figure 6C). The accuracy of CAs depends on the initial variables as well as the amount of noise in the recorded video. We discuss how a negative situation can affect CAs’ accuracy in the sensitivity analysis section in the Supporting Information.

### Samples with a Chemical Heterogeneity

**Figure 8:**
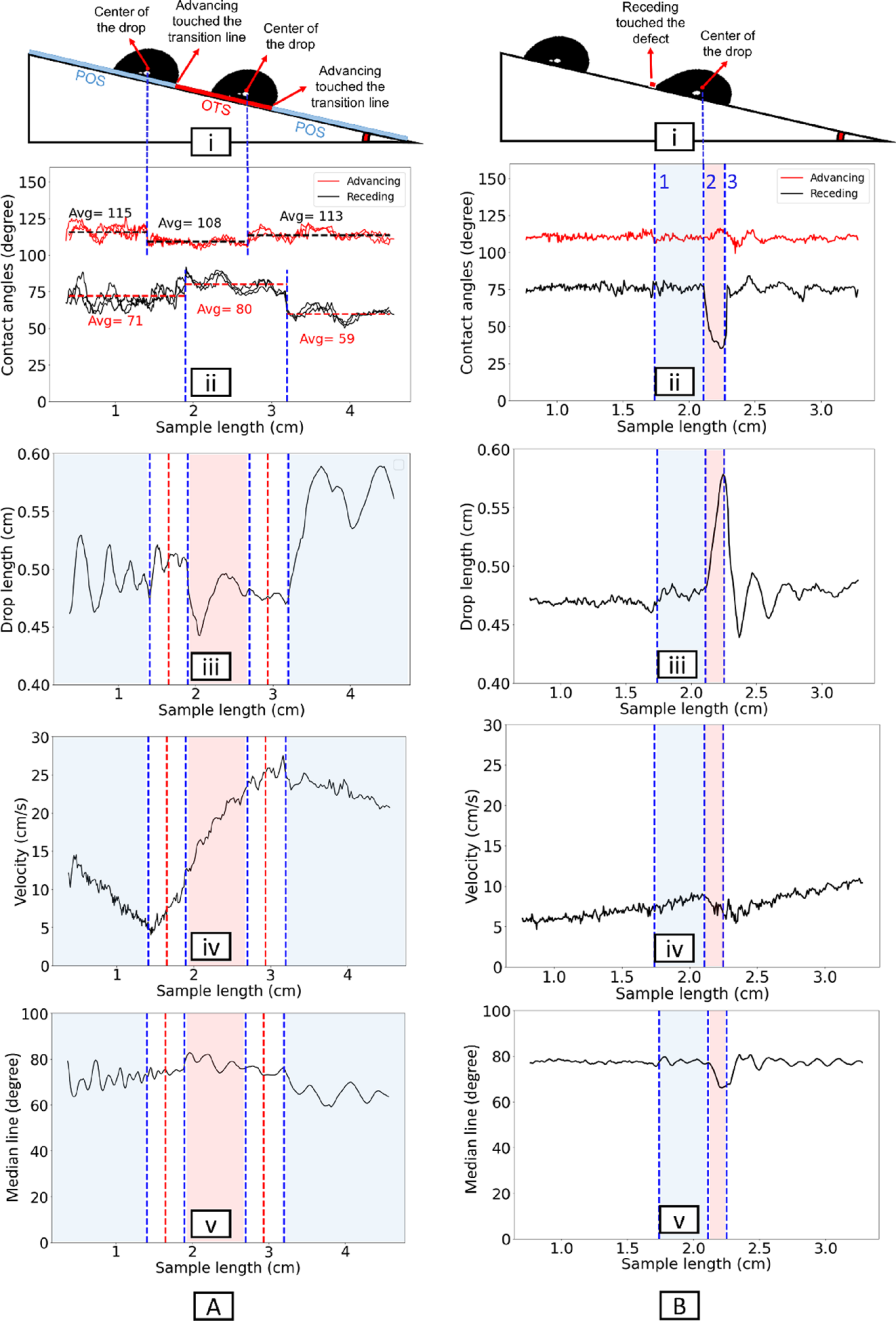
The drop profile when it is sliding on a sample with a defect and a sample with a chemical heterogeneity. Column A; A_i: The sliding drop on a sample with a chemical heterogeneity. A_ii: The CAs’ diagram based on the sample length. The first and third blue lines indicate when the advancing part touched the new surface, and the second and fourth blue lines indicate when the receding part touched the new surface. The red line represents the exact position of the transition line calculated from the blue lines and drop length. The blue area represents when the drop is completely on the POS, while the red area represents when it is completely on the OTS. A_iii: The receding diagram based on the sample length. A_iv: The velocity diagram based on the sample length. A_v: The median line angle based on the sample length. Column B; B_i: The sliding drop on a sample with a defect. B_ii: The CAs’ diagram based on the sample length. The first blue line represents the advancing part touching the defect, the second blue line represents the receding part touching and pinning the defect, and the third blue line represents the receding part depinning from the defect. There is a blue area when the defect is inside the drop and a red area when the drop is pinned. B_iii: The drop length diagram based on the sample length. B_iv: The velocity diagram based on the sample length. B_v: The median line angle based on the sample length.

We will plot and discuss the drop’s movement on different samples in this and the next section. As the toolkit output, we are interested in the details of movement rather than its physics, which would require more experiments with different parameters. To analyze the sample with a chemical heterogeneity, we selected a tilt angle of 35° (Figure 8A_i). The tilt angle was not high enough for drops to slide on the POS surface. To accelerate the drops reaching the OTS surface, the needle and surface distance was increased to 0.5 cm. On OTS, the CA hysteresis is lower and the drop accelerates. When the advancing part reached the first transition line (*x* = 1.4 cm), θ_a_ dropped by 7° (Figure 8A_ii), and when it reached the second transition line (*x* = 2.7 cm), θ_a_ increased by 5°. The θ_r_ did not change in *x* = 1.4 cm, neither *x* = 2.7 cm. However, θ_r_ increased and decreased at *x* = 1.9 cm and *x* = 3.2 cm where the receding part touched the transition lines, respectively. The exact position of the first and second transition lines can be determined precisely by using the changing points of θ_a_ and θ_r_, considering the drop length information for each moment of sliding. Therefore, *x* = 1.64 cm and *x* = 2.93 cm are the first and second transition lines’ positions, respectively (Figure 8A_iii–v, red lines). The blue and red areas represent when the drop is completely in POS and OTS, respectively. Observing the jump of the θ_a_ and θ_r_ when they cross the transition line was more clear when we conducted three independent experiments (in Figure 8A_ii, three lines for θ_a_ and θ_r_ are plotted). A smooth change in θ_a_ and its inherent difficulty in measuring CAs beyond 90° justify using the proposed method for analyzing θ_a_. Based on standard dynamic CA measurement, we measured θ_a_ = 124° and θ_r_ = 83° for POS and θ_a_ = 113° and θ_r_ = 92° for OTS. Based on sliding drop measurement, θ_a_ and θ_r_ values behave similarly to standard measurements. There is a decrease in their values due to the velocity, especially at the end of the movement. Due to the distance between the needle and the surface, the drop length at the starting point fluctuates rapidly (Figure 8A_iii). In general, the drop length on OTS is less than POS. The drop moves slower on the POS than on the OTS when it is on the first transition line (POS to OTS). In the same way, the drop length decreases as the drop passes the second transition line. The velocity of the drop decreases on POS and increases on OTS (Figure 8A_iv). In this way, the effect of changing the surface on velocity differences can be seen clearly. As a result of needle distance and two transition lines, the drop fluctuates throughout the route (Figure 8A_v). Water drop oscillations are clearly seen to increase after passing through transition lines.

### Samples with a Topographic Defect

For the sample with defect (Figure 8B_i), we calculated the drop’s CAs of θ_a_= 108° and θ_r_= 75° (Figure 8B_ii). At first, θ_a_ plateaued with an average of 108° and a standard deviation of 2°. Similarly, θ_r_ had an average of 75°. When the center of the drop reached a sliding length of *x* = 1.75 cm (1st blue vertical line). At this position, the advancing side of the drop touched the defect and θ_a_ dropped from 108° to 101°. Then, the drop’s receding side touched the defect at a drop’s center position of *x* = 2.11 cm (2nd blue vertical line) and θ_r_ decreased from 75° to a minimum value of 39°. Then, the three-phase contact line of the drop depinned at *x* = 2.27 cm (3rd blue vertical line). Now, θ_r_ reached its previous value and remained almost constant with a regular oscillation. The blue color area indicates that the defect is located inside the drop, and the red color area signifies that the drop is pinned. Before touching the defect, the drop length diagram was plateaued (Figure 8B_iii). After the advancing part touched the defect, the drop length increased rapidly and plateaued at a higher value when the defect was inside the drop (blue color area). While the receding part got stuck when it touched the defect, the advancing part kept moving. Therefore, the drop length started to increase gradually (red color area) and, after depinning, the drop length oscillated regularly. The velocity of the drop was not sensitive to the advancing part but when receding touched the defect velocity decreased significantly (Figure 8B_iv). The slope of the line became the same as before touching the defect after passing the defect. The median line angle was quite constant before touching the defect due to the gentle placement of the water drop (there was no needle distance). When the advancing contact lines touched the defect, the median line angle started to fluctuate slightly. Once the receding part depinned from the defect, we measured stronger fluctuations in drop movement (Figure 8B_v). These fluctuations correspond to drop oscillations, which are excited by the interaction with the defect.

To analyze drop motion, we calculated the potential and kinetic energies of a drop:
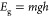
12
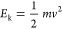
13
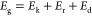
14where *E*_g_ is the potential energy, *E*_k_ is the kinetic energy, *E*_r_ is the rolling energy, and *E*_d_ is the dissipated energy.

**Figure 9:**
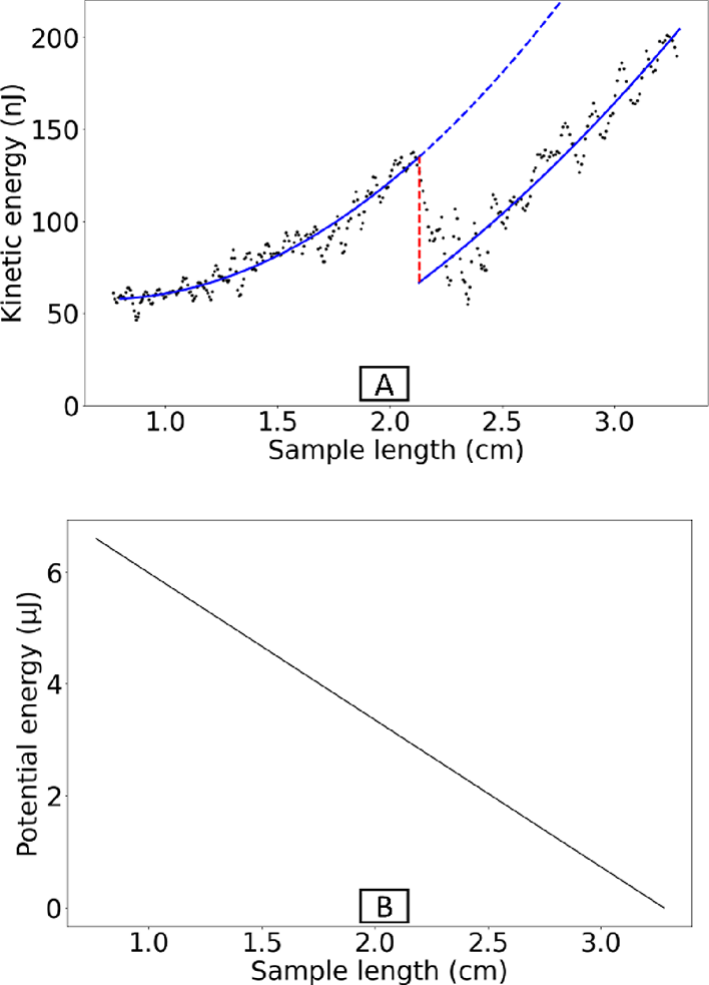
The kinetic and potential energy of the surface with the defect. (A) For each moment of movement, the black dots represent the kinetic energy. The blue lines represent the second-order polynomials fitted to the black dots. There is a difference between the two blue lines representing the defect’s effect on the energy dissipation that is marked by the red line. (B) Potential energy based on sample length.

The kinetic energy values have been smoothed by using a Savitzky–Golay filter^51^ (Figure 9A, black dots). It started from 46 nJ and increased up to 201 nJ. The kinetic energy before and after touching the defect was fitted with a second-order polynomial (due to the accelerated movement; Figure 9A, blue lines). The fitting error is about 7 nJ. Based on the difference between the two fitted diagrams (red line length), we calculated the dissipation energy due to the defect to be 68 nJ. Thus, the total energy changes in the kinetic energy diagram from beginning to end are 154 nJ, and the energy dissipation due to the defect is 68 nJ or 44%.

To compare potential and kinetic energy changes, potential energy is also calculated. The potential energy was 6.6 μJ at the beginning and decreased linearly (based on the sample length; Figure 9B). Kinetic energy changes account for only 2.3% of potential energy changes. Low velocity (high surface energy) on the surface with a defect causes this. It means that 97.7% of the potential energy dissipated or partially converts to the rolling energy based on eq 14.

The accuracy of the described criteria depends on initial variables and existing noises in the captured video. By conducting a sensitivity analysis, it is possible to determine how much a negative situation affects accuracy. The sensitivity analysis is discussed in the Supporting Information, in particular, how different criteria are affected by noises, noise removal algorithms, baseline location errors, and tilt angle measurement errors. Based on sensitivity analysis, we determined that the baseline position is the most crucial parameter for obtaining accurate CAs. Edge detection algorithms have difficulty detecting the transition line between the real drop and its reflection when the surface is transparent. The baseline detection in the transparent samples section in the Supporting Information introduces a method independent of edge detection to detect baseline in transparent samples.

## Summary and Conclusions

In order to optimize polynomial fitting and measure the accuracy, we used synthesized images. The polynomial parameters were adjusted separately for the front and rear of the drop. By finding the best values for the *n* and *p* according to the sliding drop image resolution, the accuracy based on MSE is 3.41 for angles 15–90° and 10.9 for angles 90–165°. A super-resolution model based on deep learning was developed that increased the original image 9 times with an accuracy of 36.39 PSNR. Magnified images improved the measurement accuracy as discussed. A final improvement arose from the fact that the error distribution is not the same for different angles. As a result, the angle measurement accuracy was once again improved to 2.11 for CAs <90° and to 4.91 for CAs >90°. This means that the accuracy improved by 21% for CAs <90° and 33% for CAs >90° when using a 4S-SROF.

We developed a toolkit that can automatically extract the drop profile at every moment of movement. The parameters extracted are the drop length, middle line angle, velocity, receding angle, and advancing angle. These parameters can be extracted simultaneously to help researchers conduct detailed studies with a broad range of variables taking into consideration their correlations.

## Supporting Information Available

Supporting figures, sensitivity analysis, and baseline detection for special cases (PDF)
Video showing how the toolkit works, an example of drop sliding on a sample with a topographic defect (MP4)
