# Supplementary material for: Deep Learning to Analyze Sliding Drops
Source: Langmuir. 2023 Jan 12;39(3):1111–22. doi: 10.1021/acs.langmuir.2c02847 (PMC9878717; doi:10.1021/acs.langmuir.2c02847)
Supplement: Supplementary file 1 — la2c02847_si_001.pdf [file la2c02847_si_001.pdf]

# Supporting information

# Deep learning to analyze sliding drops

*Sajjad Shumaly†, Fahimeh Darvish†, Xiaomei Li†, Alexander Saal†, Chirag Hinduja†,*

*Werner Steffen†, Oleksandra Kukharenko†, Hans-Jürgen Butt†, Rüdiger Berger*†*

† Max Planck Institute for Polymer Research, Ackermannweg 10, D-55128, Mainz, Germany

* Corresponding Author. Email: berger@mpip-mainz.mpg.de

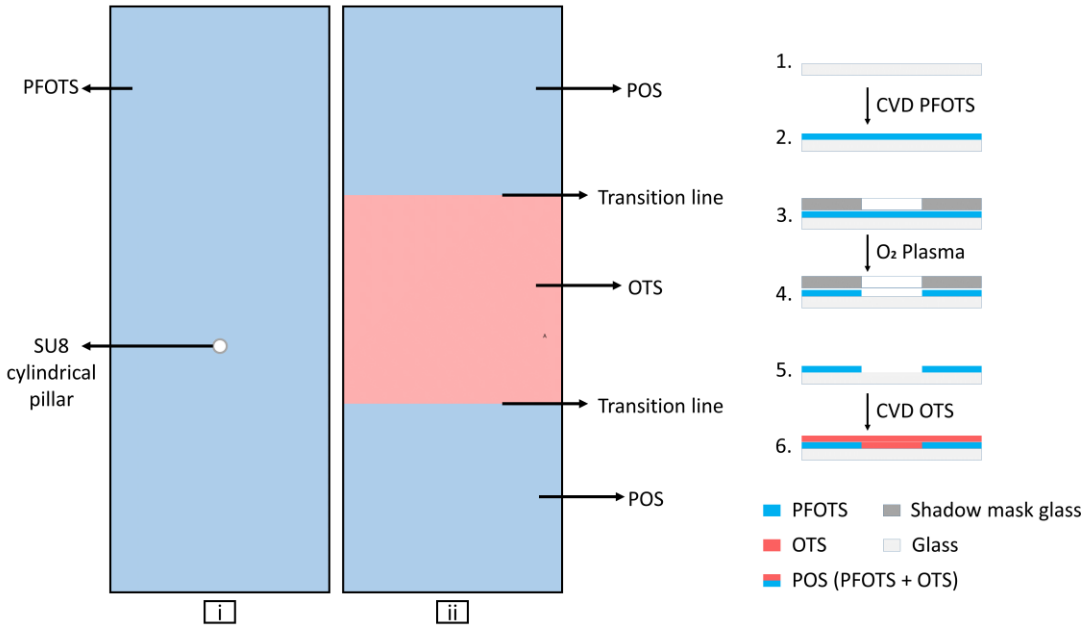


(a) (b)

**Figure S1.** A schematic showing the appearance of samples. a_i: a sample with a topographic defect is covered by PFOTS and a SU8 cylindrical pillar is in the middle of the sample. a_ii: a sample with a chemical heterogeneity contains two different areas (OTS and POS) and two different transition lines (from POS to OTS and from OTS to POS). b: steps of preparing a sample with a chemical heterogeneity are represented for a better understanding.

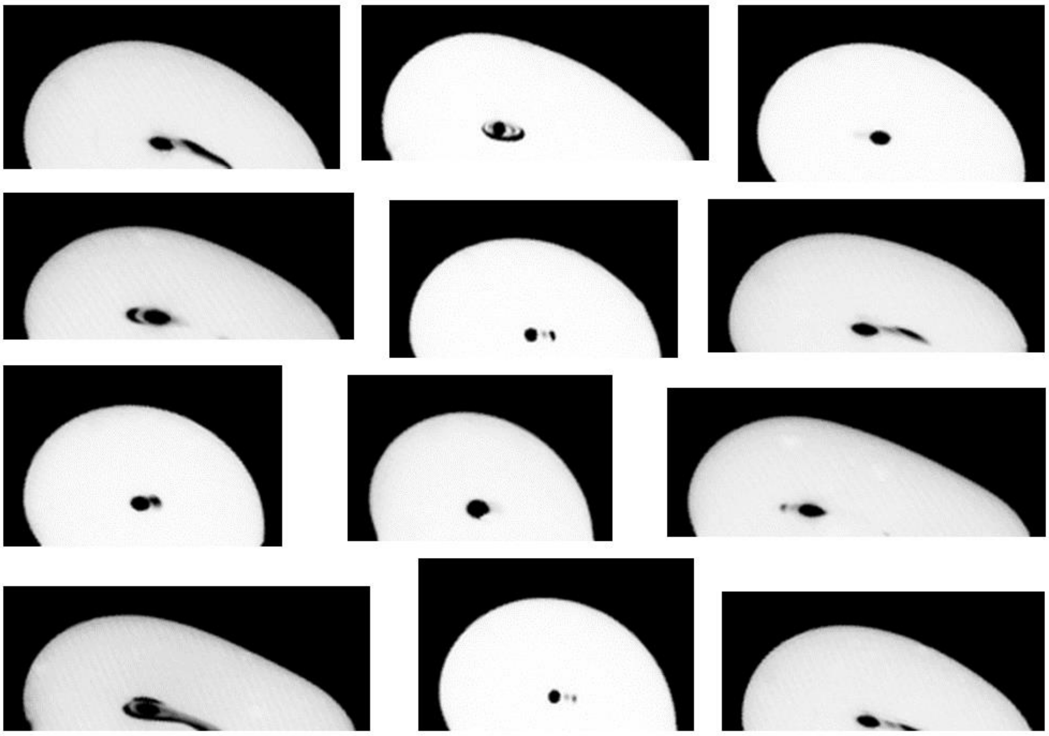

**Figure S2.** Examples of drop images in the dataset used for model training.

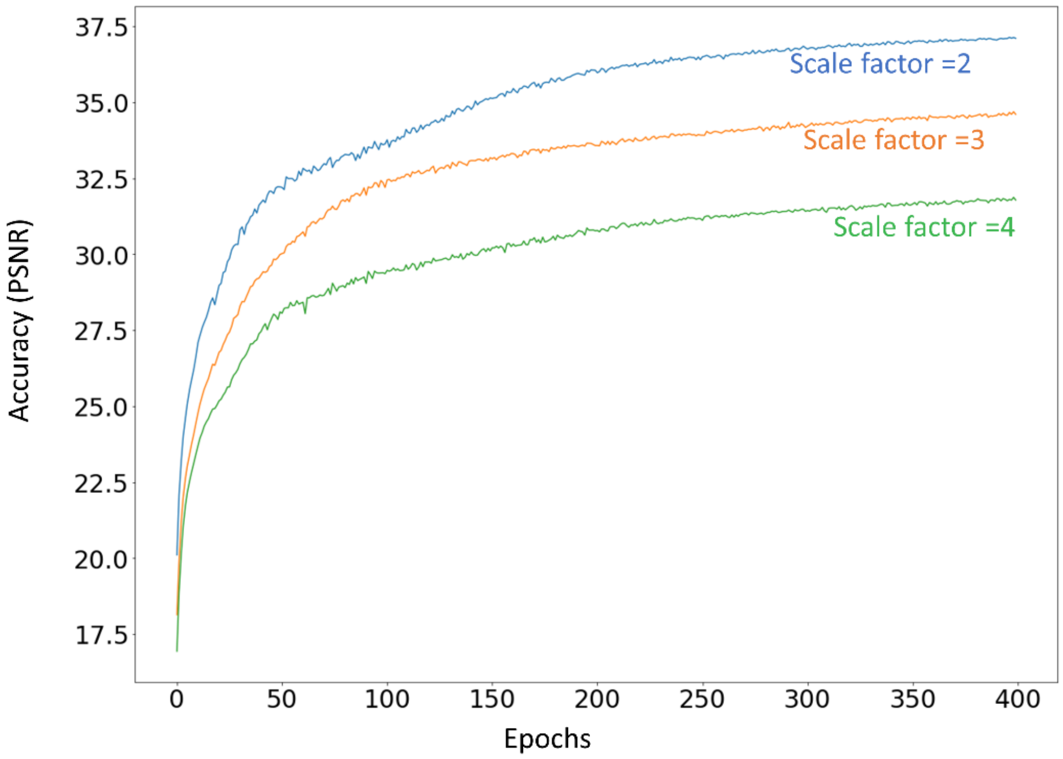


**Figure S3.** Effect of changing scale factor on accuracy diagram. We considered 50 drop images from the test set to compare these three diagrams in each epoch. The figure shows as the scale factor increases, the problem becomes more complex and accuracy decreases.

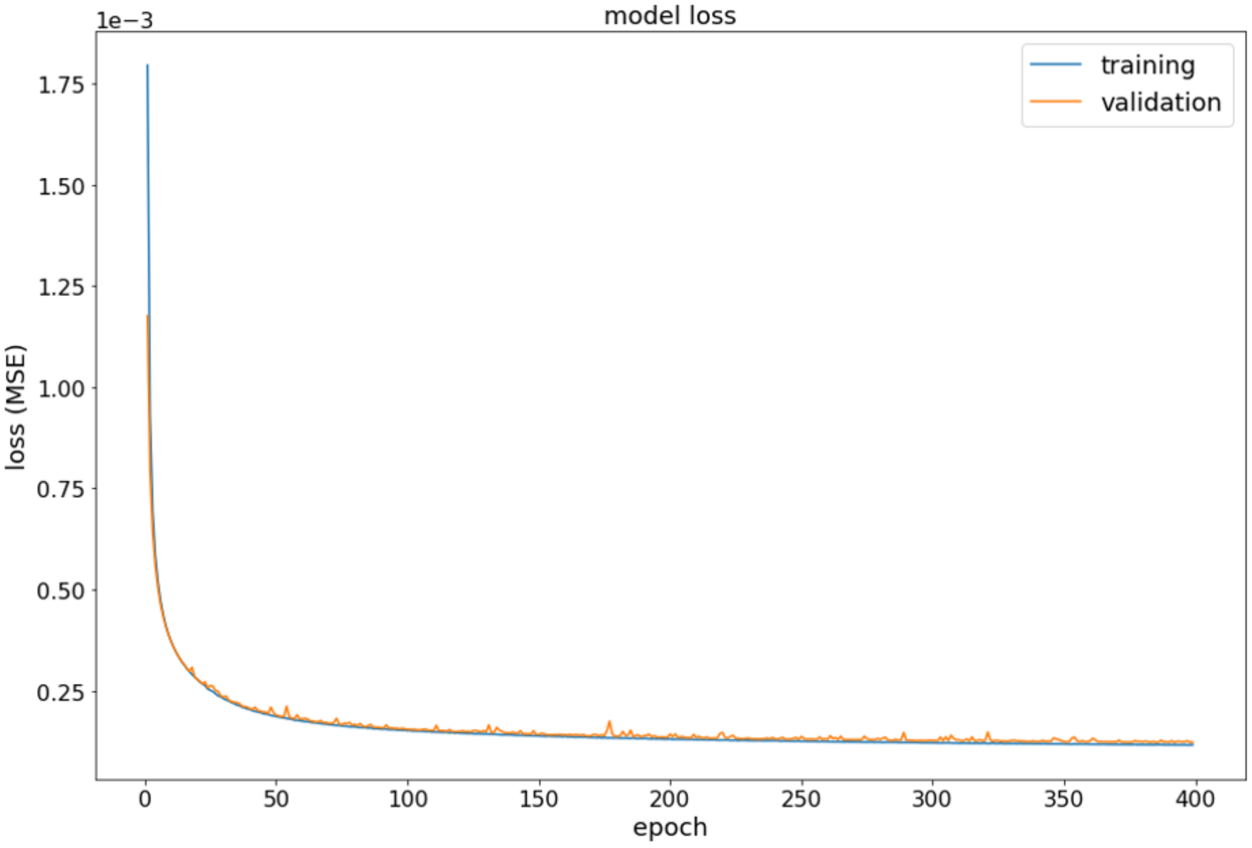


**Figure S4.** The training process of the modified ESPCN model based on MSE as the loss function.

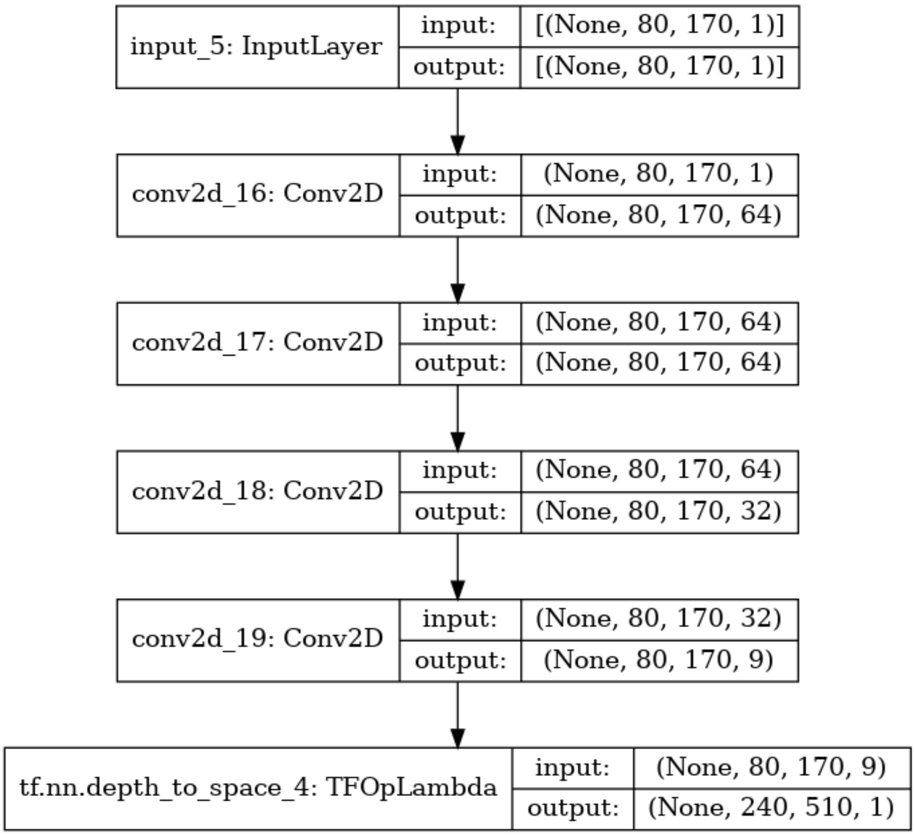


**Figure S5.** The modified ESPCN architecture.

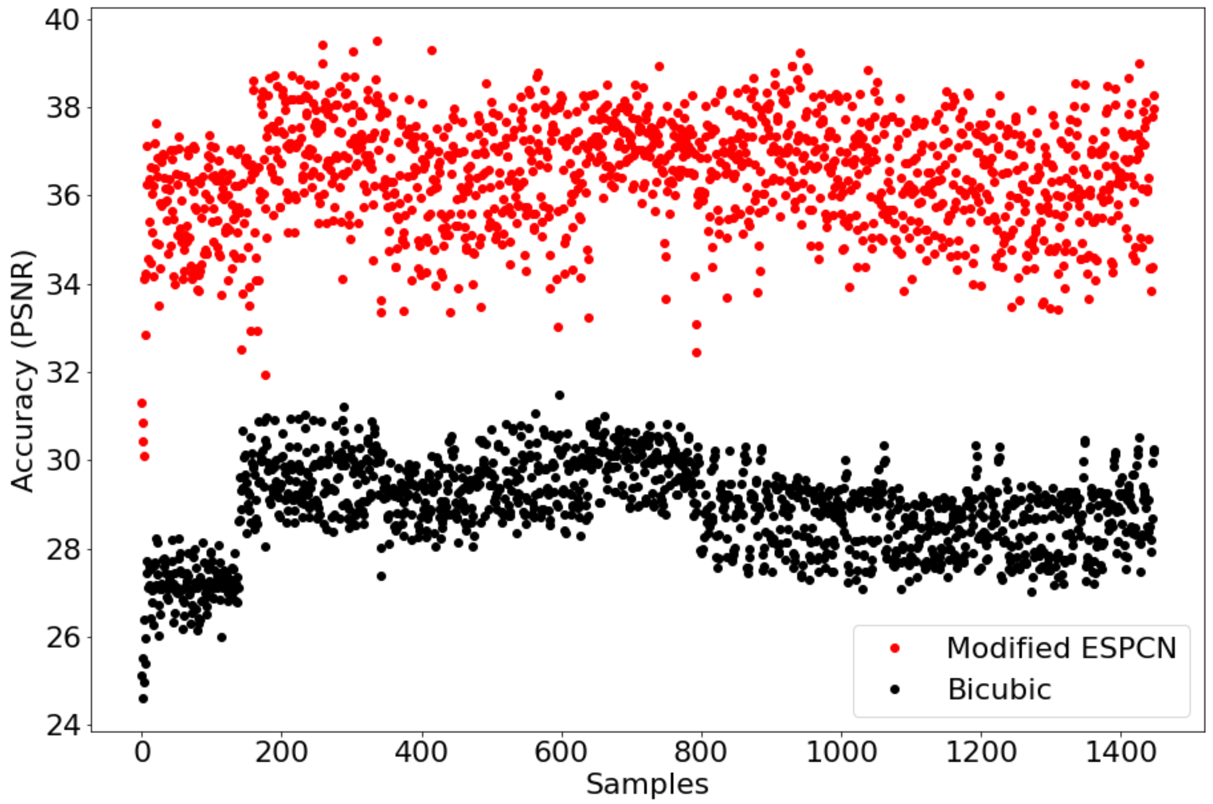


**Figure S6.** . Accuracy distribution of the Bicubic method and the modified ESPCN for the same 1400 samples of the test data set. All test samples belong to separate videos that were not used in the training process. The average PSNR for the Bicubic method corresponds to 28.90 and for the modified ESPCN to 36.39.

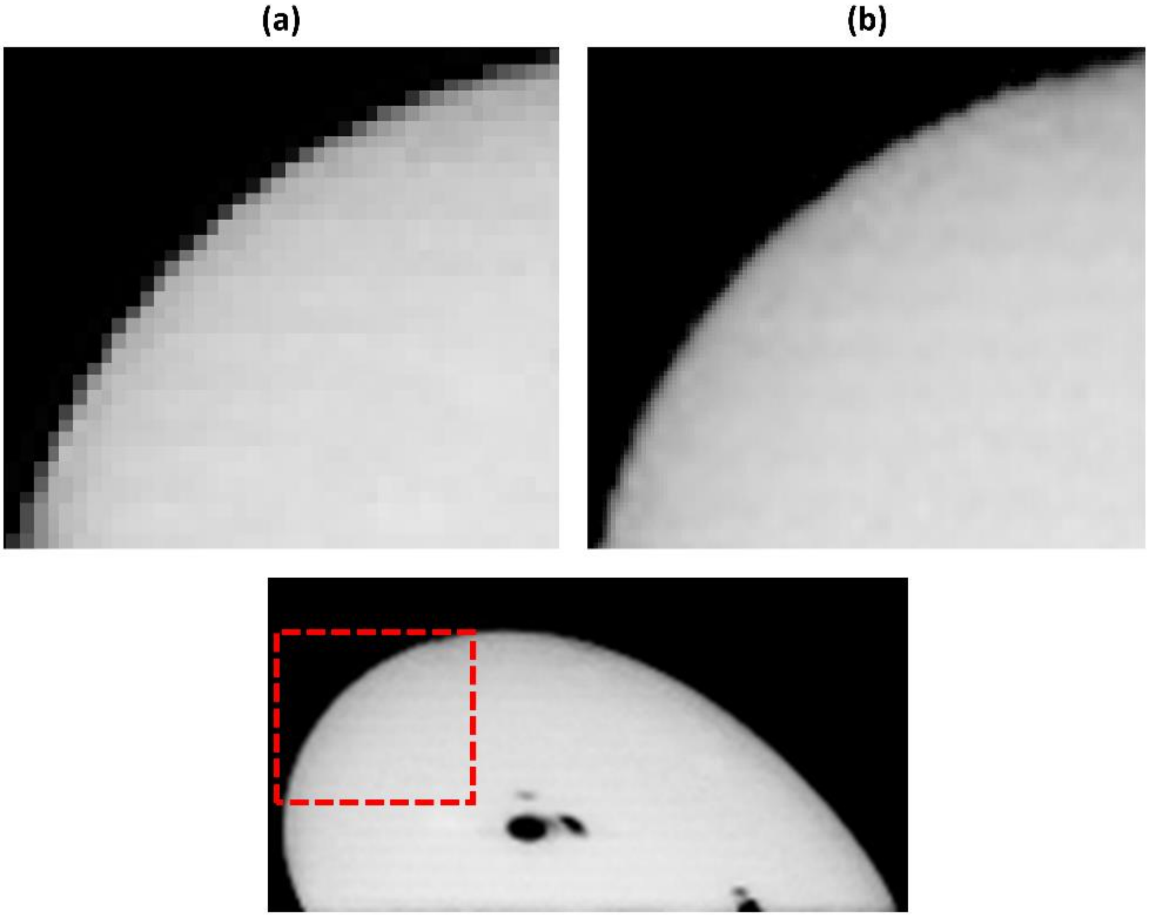


**Figure S7.** The accuracy of drop images before (a) and after (b) the super-resolution model.

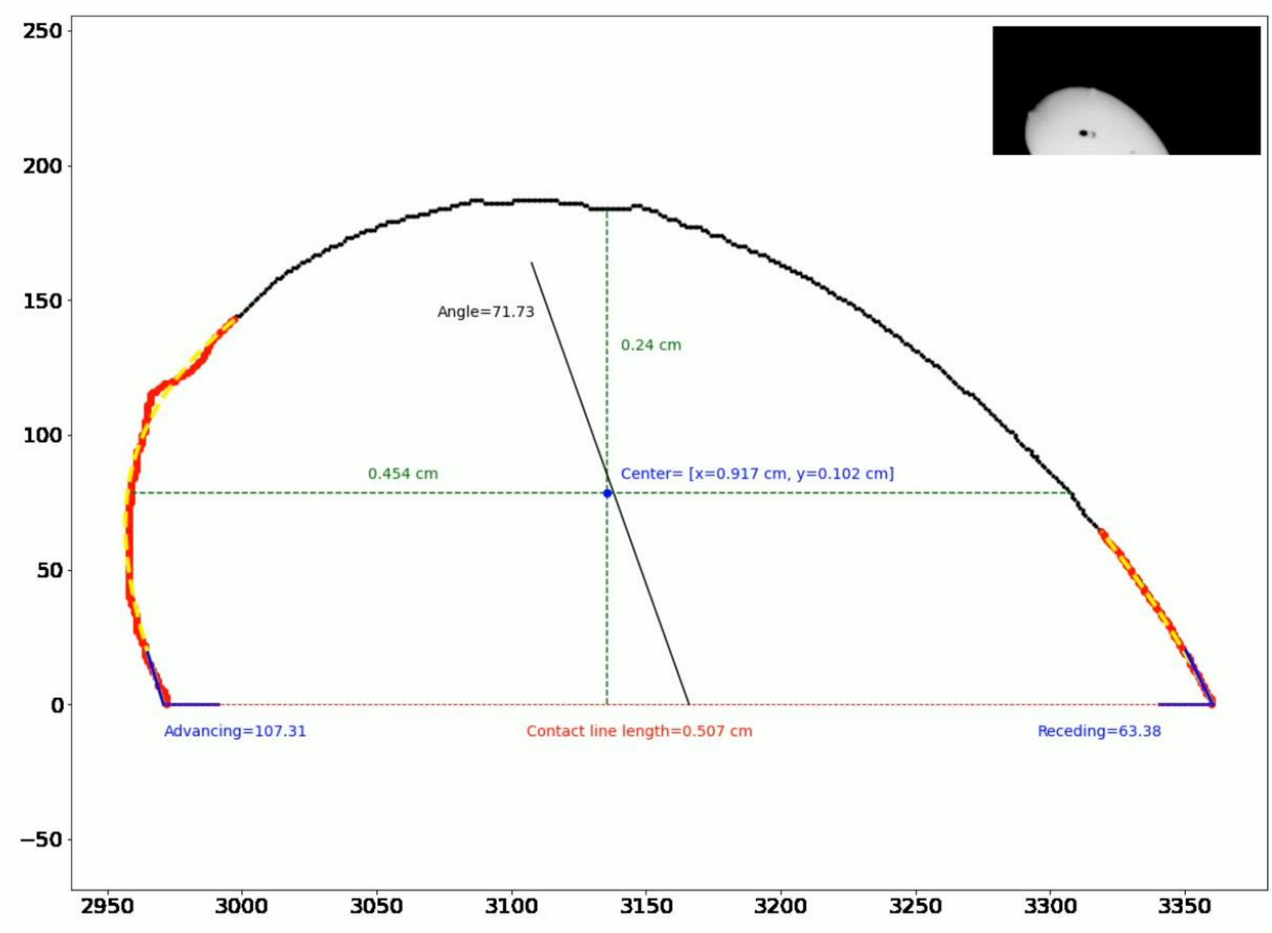


**Figure S8.** In the presence of noise, third-order polynomials can be properly generalized. Red dots represent selected pixels as input for polynomial fitting and yellow dashed represents third-order fitted polynomials. The drop edge is partially distorted but the polynomial fitting is mimicking the curve of the drop correctly.

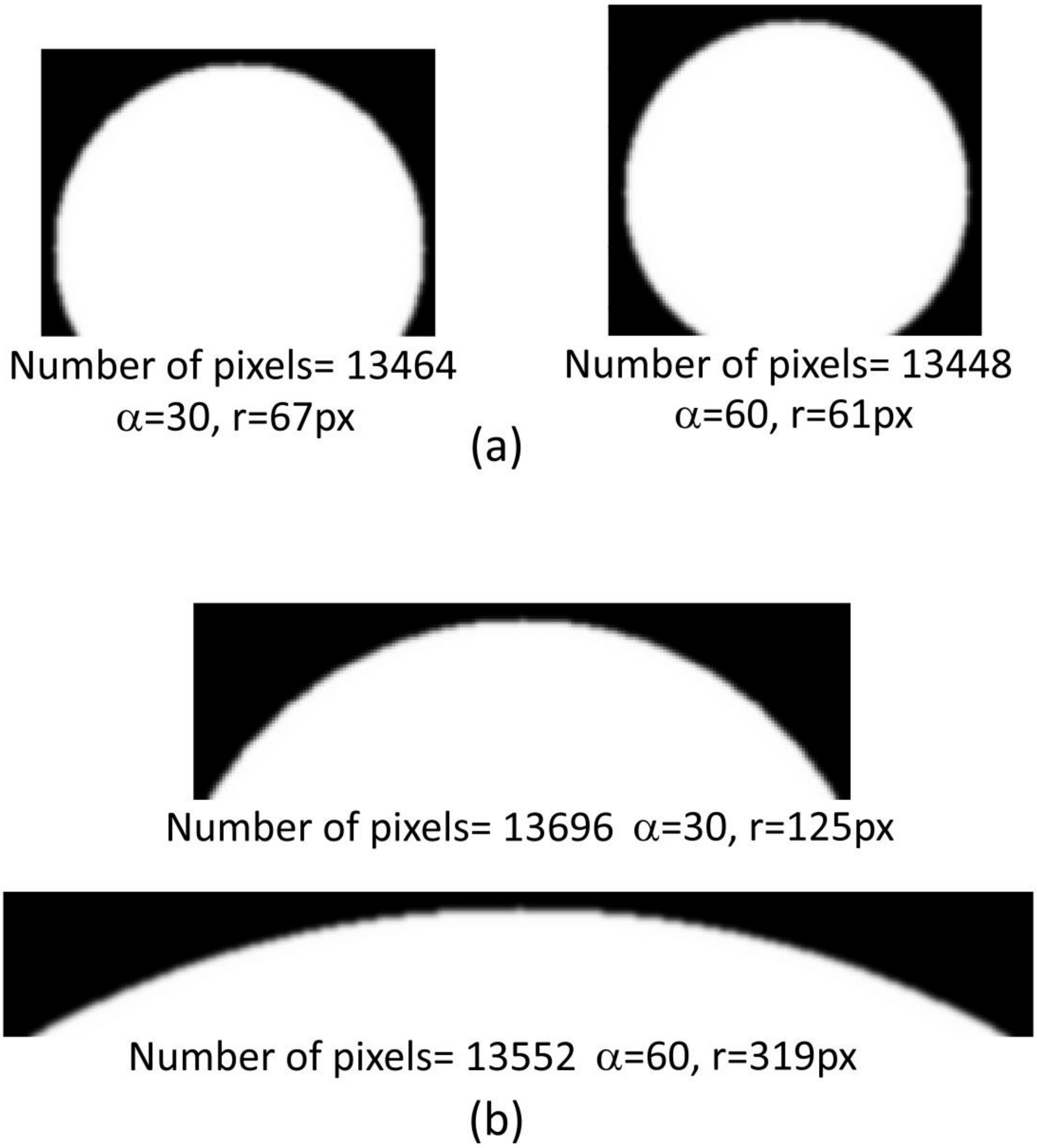


**Figure S9.** Some examples of generated drop images when α is 30, and 60 for >90° (a) and <90° (b).

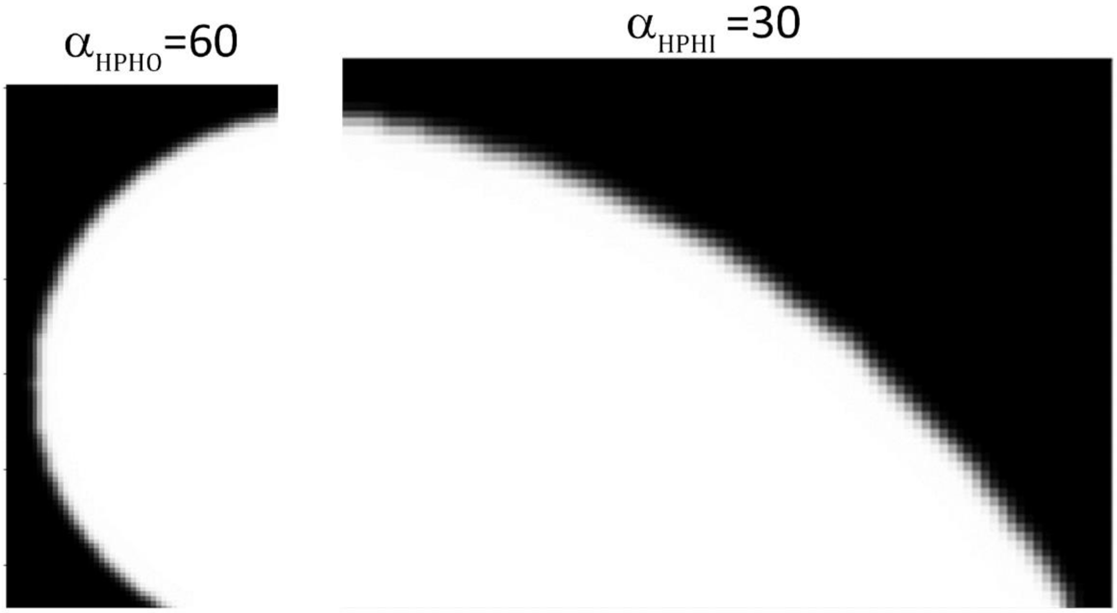


**Figure S10.** Combination of hydrophobic with α = 60 and hydrophilic with $\alpha$ = 30 synthetic images to simulate a sliding drop advancing part and receding part.

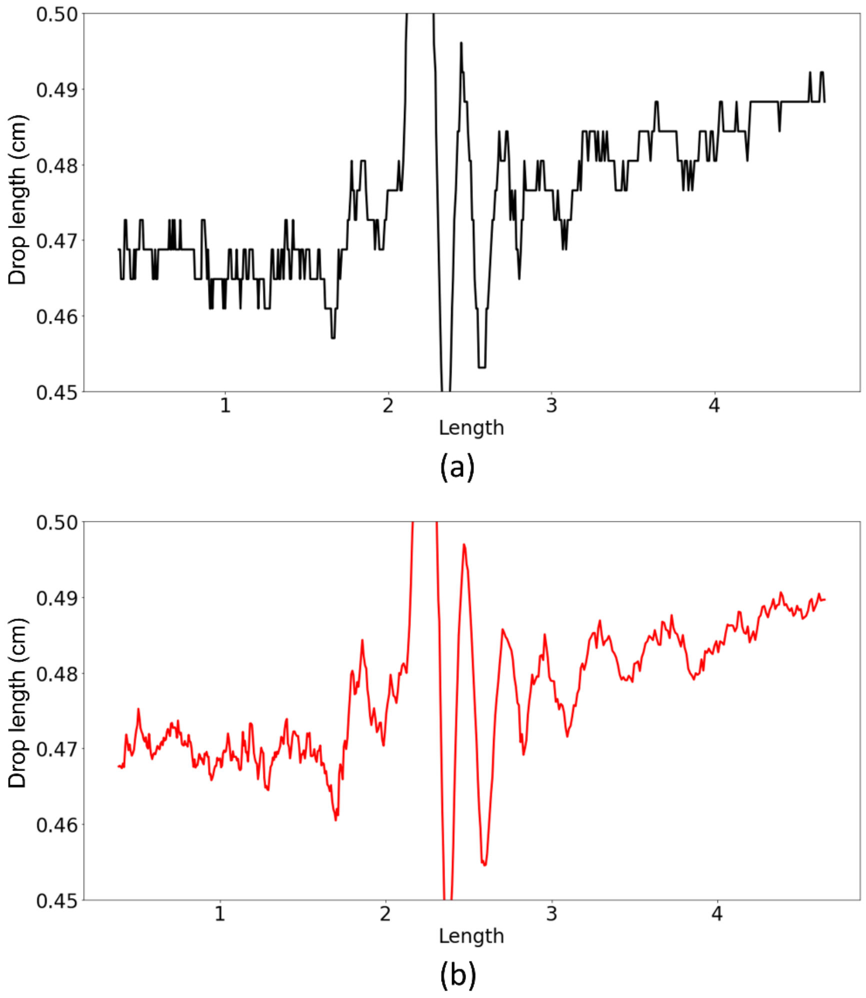


**Figure S11.** Changes in the drop length (cm) of a drop on a sample with a defect based on sample length (cm). Calculated drop length a) before using the polynomial fitting on a low-resolution image b) after using the polynomial fitting on a super-resolution image. The drop length depends on the difference between the two end pixels of the drop curve. But using the polynomial fitting, the exact location of the two end pixels of the drop curve is determined by its adjacent pixels. This approach increases accuracy.

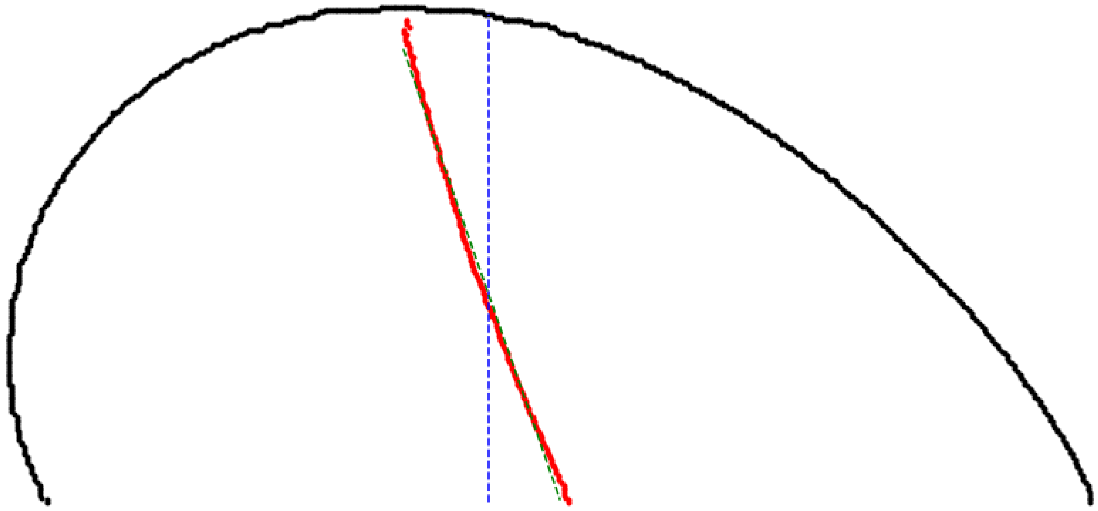

**Figure S12.** A drop contour in super-resolution space. Red dots represent the middle of each row of the image. Their shape resembles a line. The green line is a fitted line to them. This line is considered the median line. The blue line is the weighted average of the red dots. This line is used to determine the position of the drop on the sample length and velocity calculations are based on the blue line as well.

## Sensitivity Analysis

Existing noises in sliding drop videos are a serious problem that needs to be addressed. Most of the noises belong to the background of the video and removing them is not challenging. But some others may appear due to different reasons. Using noise removal algorithms can cause unwanted side effects on drop boundaries. The other issue is that drop profile extracting can be affected by errors in initial variables, such as baseline location and tilt angle. This section discusses the sensitivity of the sliding drop problem to noises, noise removal algorithms, baseline location errors, and tilt angle measurement errors.

### Noise handling

Noise can sometimes be distinguished by drop boundaries. Some techniques like median filters can be used to remove noise in this situation. But, noises may be very close to drop boundaries and difficult to detect. The maximum effect they have on CAs values will be when they are close to the baseline. An advancing diagram related to a sliding drop video may be influenced significantly by noises (Figure S13). Figure S13_1 represents a normal sliding drop image without any noises. But sometimes there are noises inside the taken image. In some cases, the noise is close to the drop edge (Figure S13a_2). Removing this type of noise is not possible and drop boundaries will be damaged. In this case, noise is a part of the drop and distinguishing them is not possible based on image processing algorithms. But, after extracting the drop profile and contact angles, a big leap represents noise that can be removed using signal processing techniques. In other cases where the noise has not disturbed the edge of the drop, it can be removed by image processing methods (Figure S13a_3).

We studied noise removal algorithms' effects on an advancing diagram (Figure S13b). A median filter with a kernel size of 3 and a morphological transformation by kernel size of 4 was considered a noise removal algorithm. The median filter works well at removing the noise of the 3rd drop, but it was not able to follow the original diagram in some areas and some

displacement occurs (Figure S13b, yellow asterisks). The morphological transformation works based on erosion followed by dilation. This algorithm removed the noise of the 3rd drop and followed the original diagram better than the median filter. The noise removal algorithms could not remove the noise of the 2nd drop.

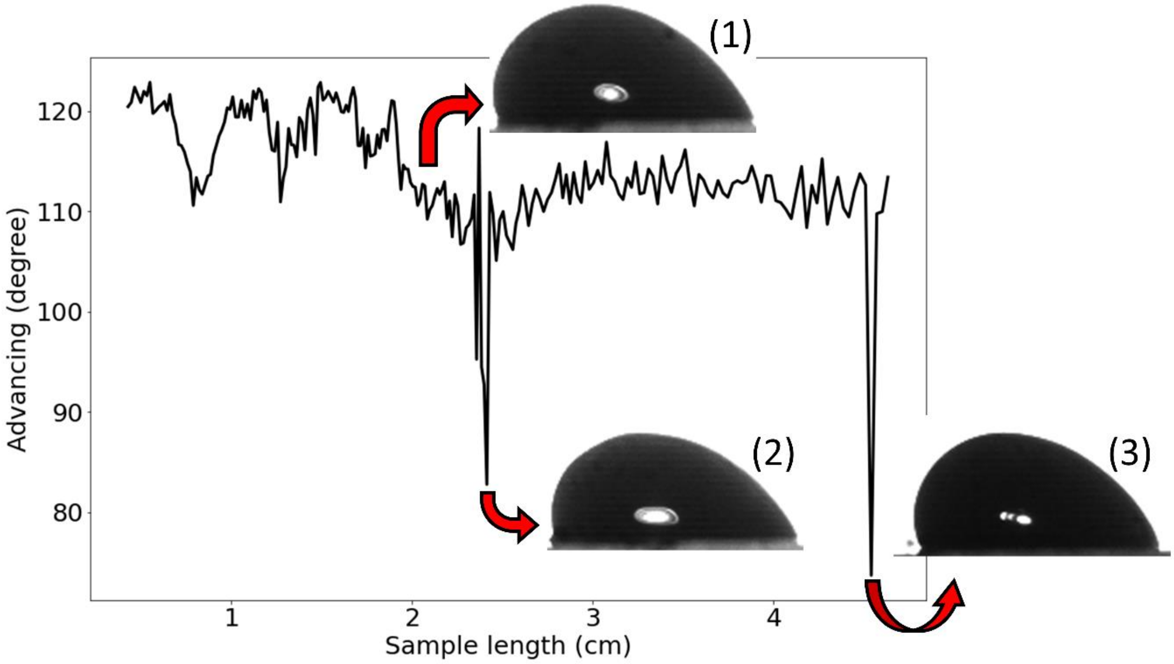


(a)

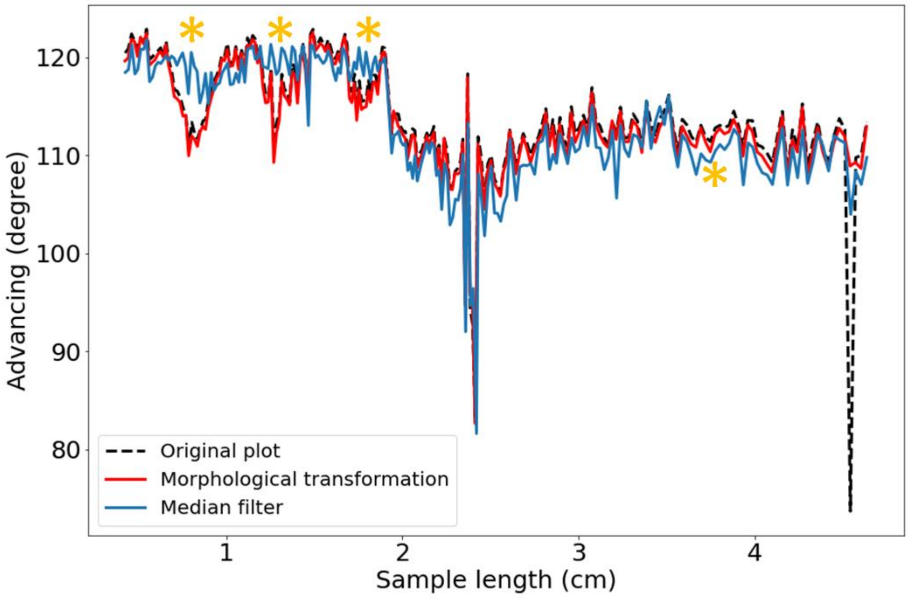


(b)

**Figure S13.** The effect of noise removal algorithms on the advancing diagram. a) Advancing diagram related to a sliding drop video, a_1: A normal drop image without any noise, a_2: A drop image with a noise close to the drop edge, a_3: A drop with a detectable noise. b) The black dotted plot is the original plot before using noise removal algorithms. The blue line is the advancing diagram after using the median filter method with a kernel size of 3. The red line is

the advancing diagram after using the morphological transformation method with a kernel size of 4. Both the median filter and morphological transformation are able to remove the 3rd noise. But the median filter may change the drop edge and CA diagram in some cases. The yellow asterisks represent displacement caused by the median filter.

The proposed drop video analysis toolkit uses the morphological transformation method. However, morphological transformation does not come without side effects. The receding part of the drop is usually the most sensitive to this method. Erosion may detect the very end of the receding part as noise when receding is very low. It happens when the kernel size is large (Figure S14a). The larger the kernel size, the greater the displacement. However, the smaller kernel sizes can eliminate most of the noises and keep the receding value close to the original value (Figure S14b). In the proposed program default kernel size is considered 3.

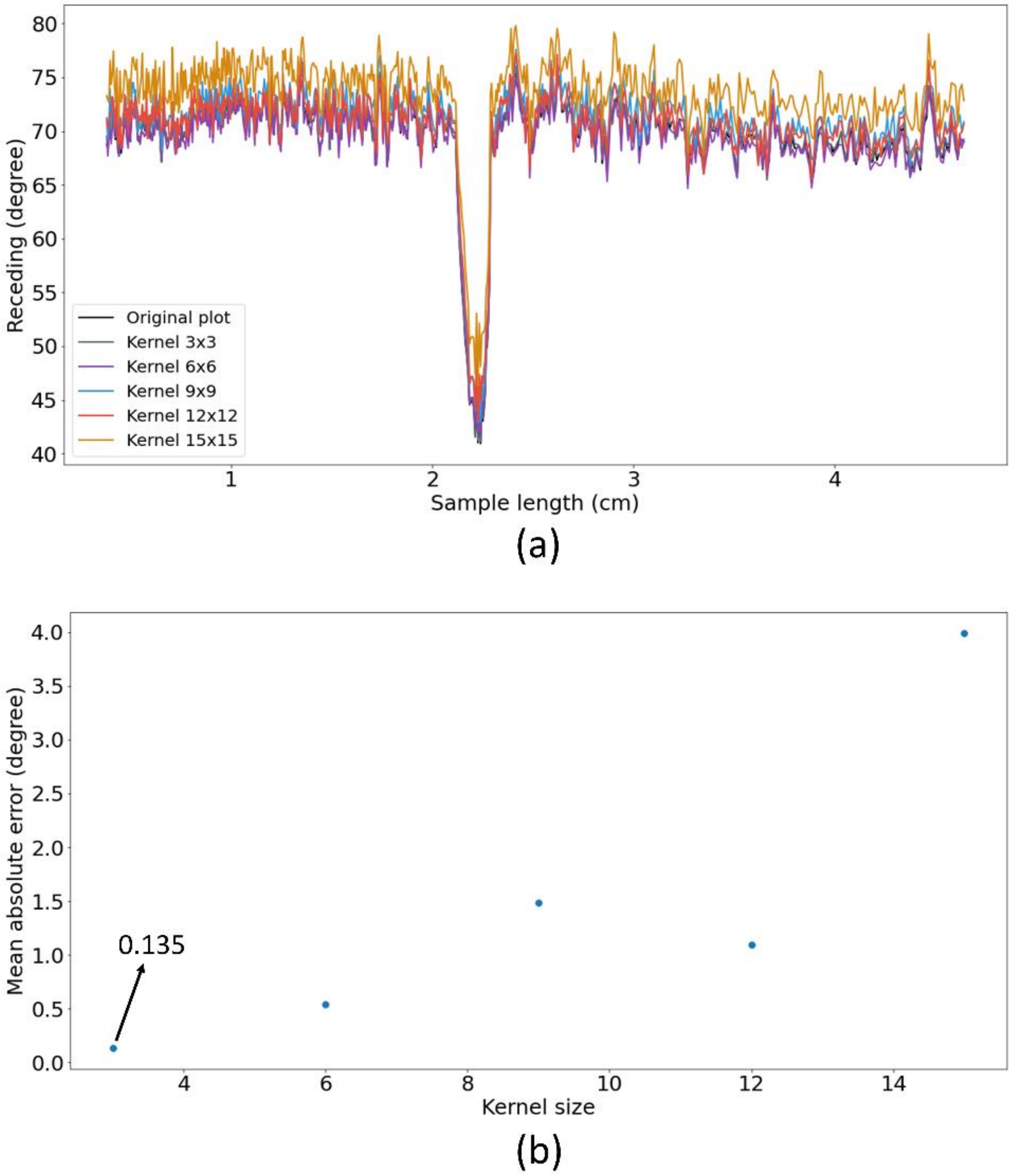


**Figure S14.** The effect of morphological transformation kernel size on the receding diagram. a) Visualizing how different morphological transformation kernel sizes affect the receding angle. The larger the kernel size, the greater the displacement. b) The morphological transformation effect on receding based on mean absolute error and kernel size. Kernel size 3 results in an MAE of only 0.135, which is very small and efficient.

**Baseline and tilt angle displacement**

Defining the exact value of baseline and tilt angle is crucial to analyzing video correctly. The sensitivity of the drop profiles such as velocity, drop length, advancing angle, and receding angle to these two parameters are studied (Figure S15). Low-value displacements of baseline and tilt angle do not affect velocity and drop length. If the error in determining the baseline and tilt angle increases, the velocity and the drop length may also be affected. In the context of advancing and receding angles, existing error in baseline and tilt angle values is influential. Displacement in baseline, 2 rows, can shift the whole of the advancing angle and receding angle diagrams up to 5° values up or down. Also when 0.2° displacement happens in tilt angle, advancing angle and receding angle diagrams in some parts may be affected up to 5°.

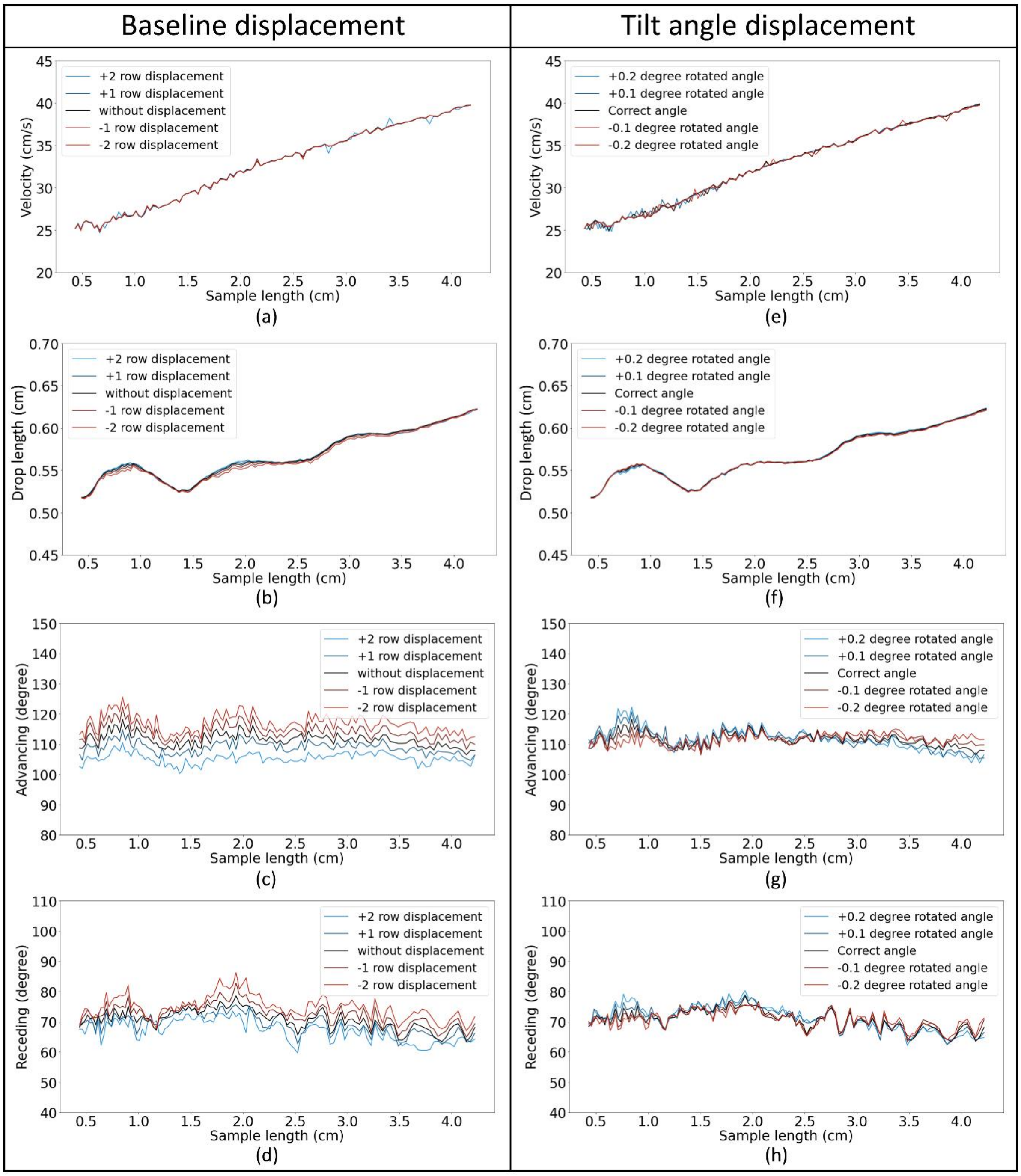


**Figure S15** A visualization of baseline and tilt angle displacement and their effect on drop profile. The effect of baseline displacement on velocity (a), drop length (b), advancing angle (c), and receding angle (d). The effect of tilt angle displacement on velocity (e), drop length (f), advancing angle (g), and receding angle (h)

## Baseline detection

A parameter that is important for extracting the CAs is the position of the baseline. Existing methods for determining baseline location are based on edge detection algorithms [1, 2]. In most cases, the receding side of the drop had not a sharp reflection. Therefore, the baseline location at the receding side is not clear. To locate the baseline, we used only the advancing side. When the surface is transparent, edge detection algorithms have difficulty detecting the transition line between the real drop and its reflection (Figure S16). It happens because the reflection of the drops on the transparent surface does not have enough contrast. Consequently, the shape of the reflection does not exactly match that of the drop, and using edge detection methods can be misleading.

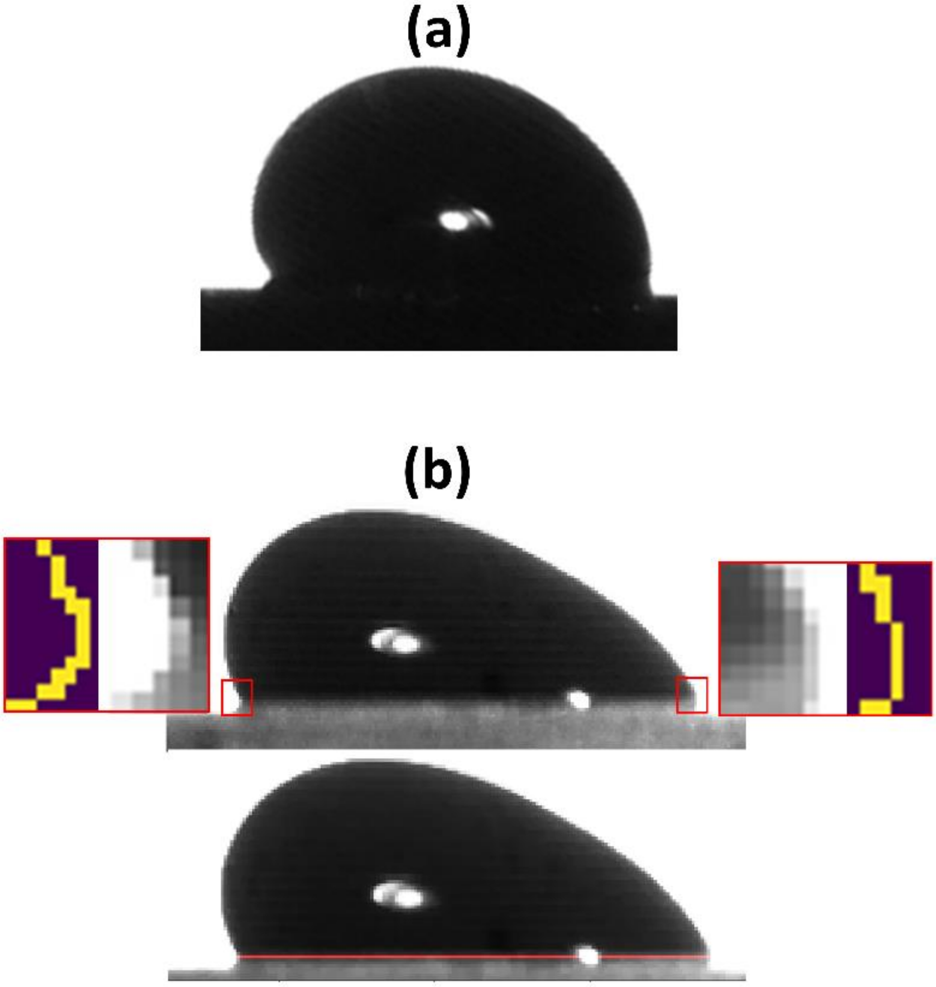


**Figure S16.** Baseline detection of drops on transparent and non-transparent samples: a) A non-transparent sample with a clear baseline. With this kind of sample, edge detection algorithms can easily find the baseline. b) The drop baseline on transparent samples is not distinct enough to be detected by edge detection algorithms. This image shows the detected edge using the canny method. No specific pixel represents the baseline in detected edges. The color-based proposed method can be applied in this case. The detected baseline (red line) is the output of the proposed method.

Here, we employ a new approach to detect the baseline on transparent surfaces, which is not based on edge detection algorithms. We analyze the intensity of pixels in our images, which is a value between 0 and 255 (0 represents black and 255 represents white). We exemplarily explain our procedure with an image showing the bottom of a drop and parts of the sample surface (Figure S17a). In this image, the drop appears white and thus has composed of pixels with intensity values close to 255. We calculate the average intensity values inside each row of the matrix (Figure S17b). Rows 1 to 20 show a slight increase in intensity due to an increase in drop size. From rows 20 to 35, there is a significant decrease in intensity and then the intensity plateaus. The latter indicates the mirror image of the drop exhibiting an almost constant but lower average intensity. The baseline is in the transition and in order to define its position we calculated the intensity derivative (Figure S17c). We attribute the minimum value of this plot to the baseline of the drop. In order to verify our procedure, we calculated the baseline separately for every frame of a video that contains 175 frames. On average, the standard error for baseline detection was 0.44 pixels, i.e. less than one pixel. Thus, the approach seems robust and from each video we can extract a reliable and constant baseline.

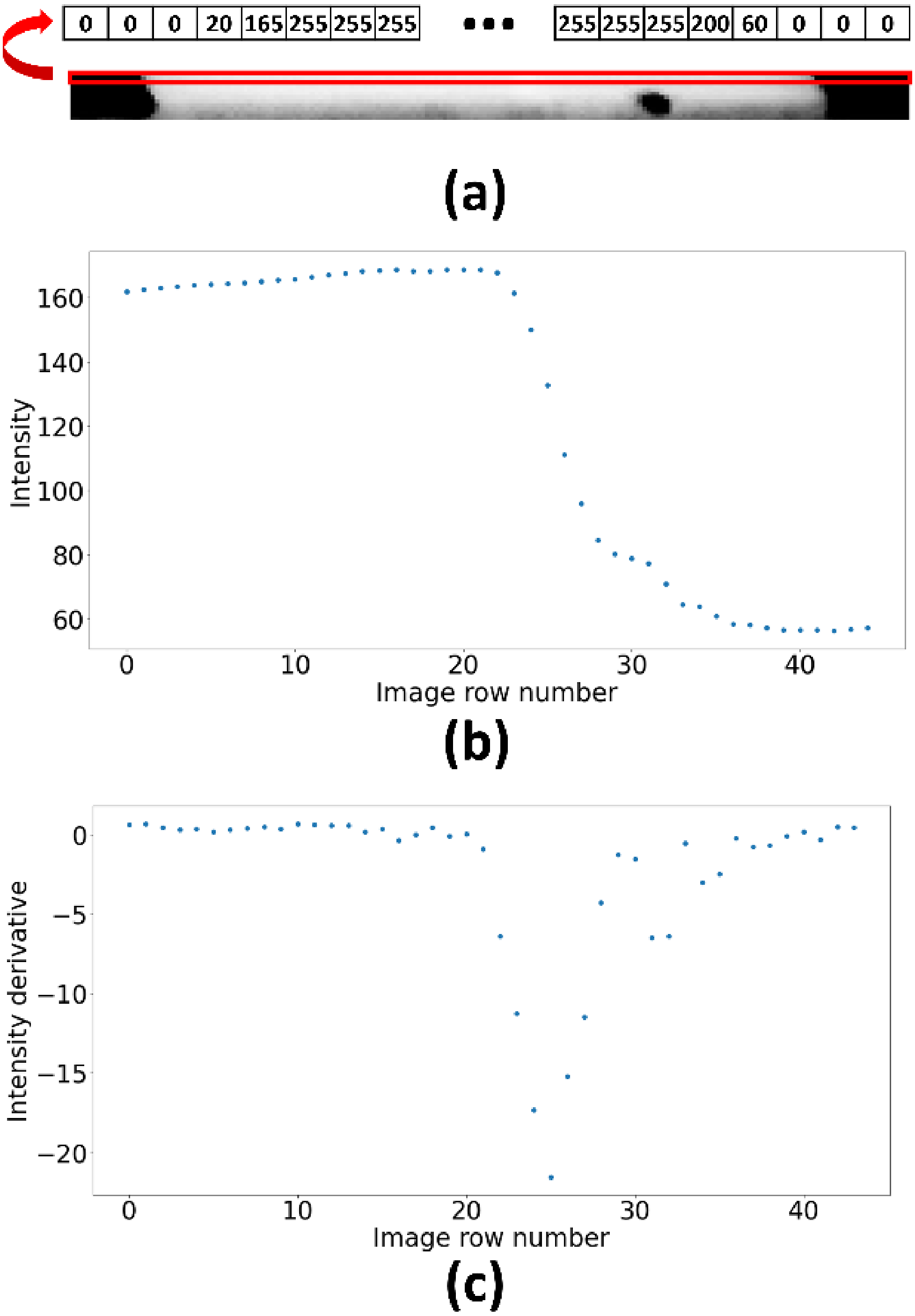


**Figure S17.** a) A representation of the first row of the numerical matrix of the drop image b) Average pixels intensity for each image row is decreasing in a specific area due to entering from the drop part to the reflection part. c) Derivative of Average pixels intensity for each image row with a minimum peak in the baseline position due to changing the environment.

## Measuring sphere diameter using super-resolution

We used images of a 5 mm diameter sphere that has a diameter similar to a drop with volume of 35 µl (Figure S18a). A standard deviation of +/- 1.6 µm was obtained after repeating the measurement ten times. The sphere was placed in the middle of the sliding drop setup and an image was taken (Figure S18b). After that, we treated the image with the Bicubic and our modified ESPCN procedure. For both procedures, we calculated the edge using a canny edge detection filter (Figure S18c). Then, we kept only the outer pixels of the sphere's edge horizontally, which is why the upside of the sphere is not connected. For further analysis, we calculated 20 times the diameter for the Bicubic and modified ESPCN model (Figure S18c). The average diameter for the Bicubic evaluation method was 5050 +/- 16.6 µm. The average diameter for the modified ESPCN evaluation was 5015 +/- 6.7 µm. The modified ESPCN has a less standard deviation.

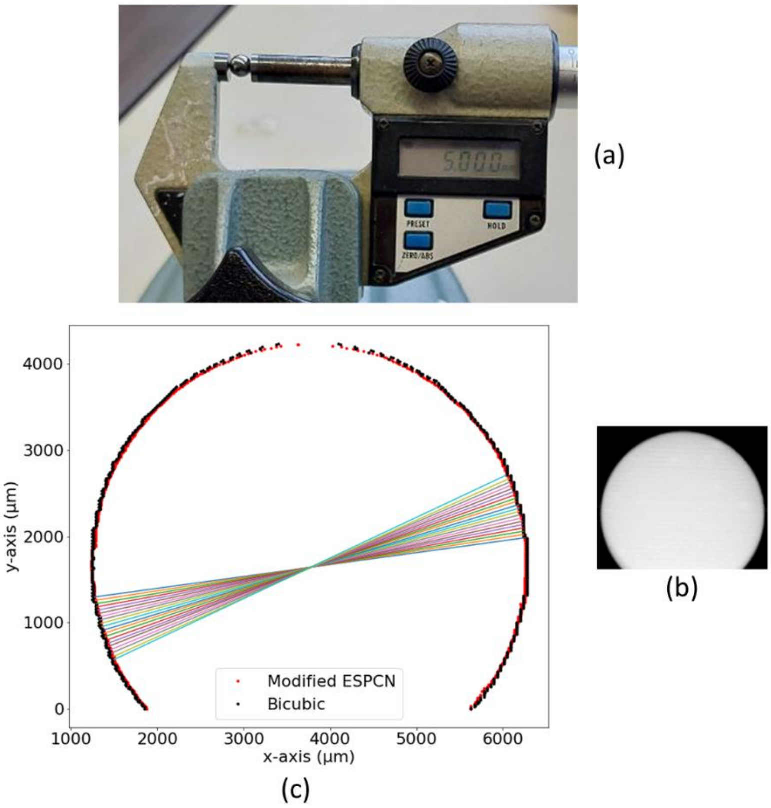


**Figure S18.** Sphere diameter measurement using Bicubic and super-resolution methods. a) Measuring real diameter of the sphere b) Sphere image in sliding drop setup c) Measuring sphere diameter 20 times using Bicubic and super-resolution methods.

## Supporting video description

The video shows a sliding drop on a sample with a defect in the middle. The first drop in the upper part of the video is a real drop image after preprocessing steps. The main steps of preprocessing included calculating the tilt angle and making the frames horizontal, removing noises and background, and detecting drop position (red lines). The second drop image which is bigger than the first one is the drop image after using a super-resolution model. Below that, the drop contour is extracted and different parameters including CAs, drop height, and drop length are displayed. On the left, four figures are getting plotted to analyze how a drop slides on a sample with a defect.

## Corresponding Author

Rüdiger Berger, Max-Planck-Institut for Polymer research (MPI-P), Ackermannweg 10, 55128 Mainz, Germany. Email: berger@mpip-mainz.mpg.de

## References

1. Kalantarian, A., R. David, and A.W. Neumann, *Methodology for High Accuracy Contact Angle Measurement.* Langmuir, 2009. **25**(24): p. 14146-14154.

2. Atefi, E., J.A. Mann Jr, and H. Tavana, *A robust polynomial fitting approach for contact angle measurements.* Langmuir, 2013. **29**(19): p. 5677-5688.
